# Supplementary material for: Whole-genome sequencing for an enhanced understanding of genetic variation among South Africans
Source: Nat Commun. 2017 Dec 12;8:2062. doi: 10.1038/s41467-017-00663-9 (PMC5727231; doi:10.1038/s41467-017-00663-9)
Supplement: Supplementary file 1 — Supplementary Information [file 41467_2017_663_MOESM1_ESM.pdf]

Description of Supplementary Files

File Name: Supplementary Information
Description: Supplementary Figures, Supplementary Tables, Supplementary Notes and Supplementary References

File Name: Supplementary Data 1
Description: P-values for differences in total ROH lengths of individuals from all 49 populations (estimated obtained using Mann-Whitney test).

File Name: Peer Review File

Suppementary Figure 1: (a) A brief representation of the analyses performed and the datasets used**.** "*" denotes KGP Phase 3; "#" denotes AGVP; "$" denotes dbSNP and "@" denotes Schlebusch et al. 2012. May et al. 2013 and other published datasets are detailed in **Supplementary Table 10**. (b) Transition-transversion ratios binned by minor allele counts (c) Venn-diagram representation of overlap between the SNPs identified using the three variant calling approaches. Overlap for four individuals (one SOT, one COL and two XHS) are shown. The patterns of overlap for the other 20 individuals were very similar to these four. The three analyses have been named Wits, Illumina and UP based on the three sites where the analyses were performed.

a.

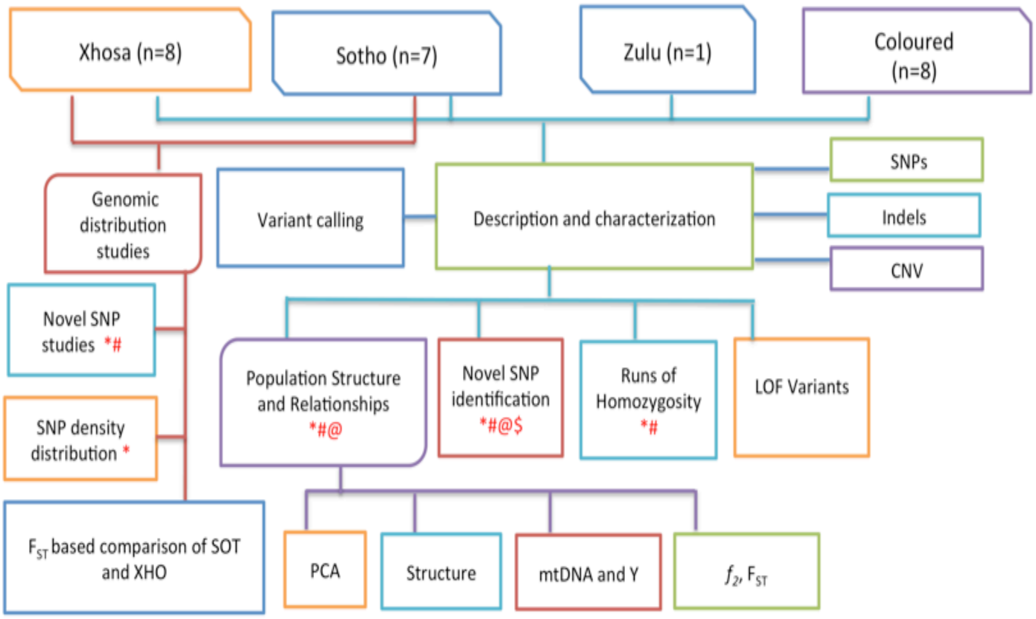

b.

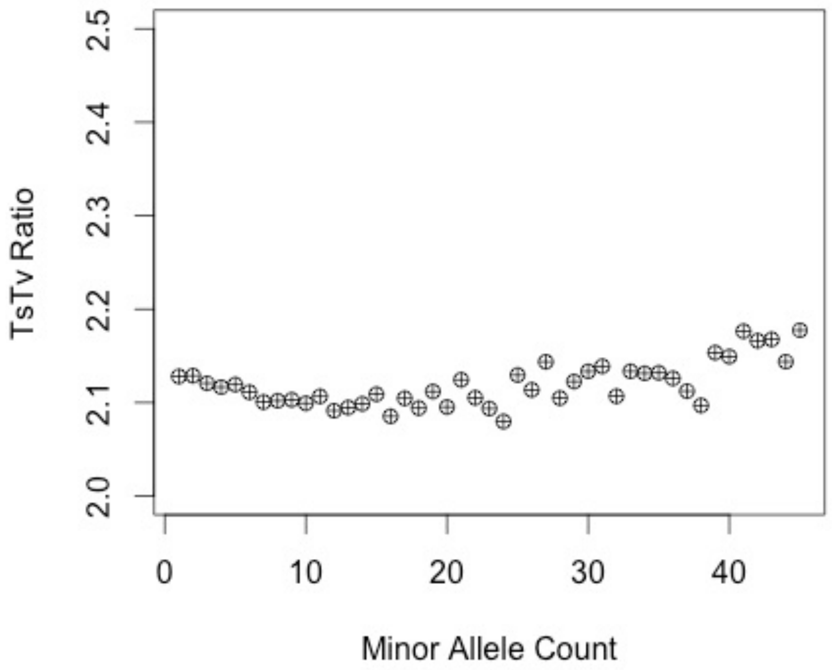

TsTv Ratio
2.0
2.1
2.2
2.3
2.4
2.5
0
10
20
30
40
Minor Allele Count

c.

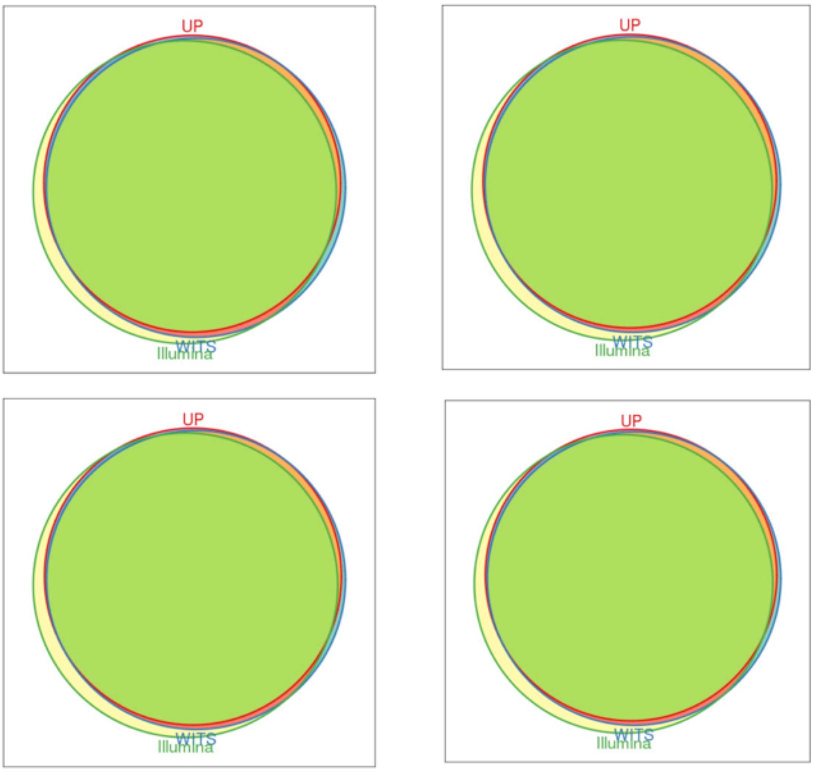

UP
WITS
Illumina
UP
WITS
Illumina
UP
WITS
Illumina
UP
WITS
Illumina

Supplementary Figure 2: Summary of distribution of Loss of function (LOF) variants. Of the 3936 LOF variants identified, only 1703 showed minor allele frequencies lower than 0.01 in other datasets (KGP and AGVP), or were novel to this study. The distribution of 1703 potentially relevant LOF variants according to functional classification (upper left) and total number of LOF variants in each individual (upper right) are shown. In the upper right plot the COL individuals are shown in light blue, ZUS in orange, SOT and green and the XHS are shown in red. 146 genes were found to contain two or more LOF variants. In 22 of these genes, we observed a possible knockout configuration (one LOF variant on each chromosome).

Type	Number of variants
Stopgain	370
Stoploss	46
Frameshift insertion	302
Frameshift deletion	534
Splicing	459

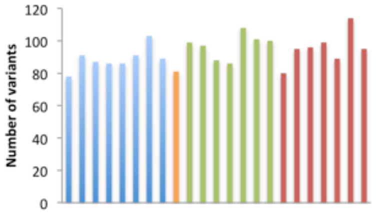

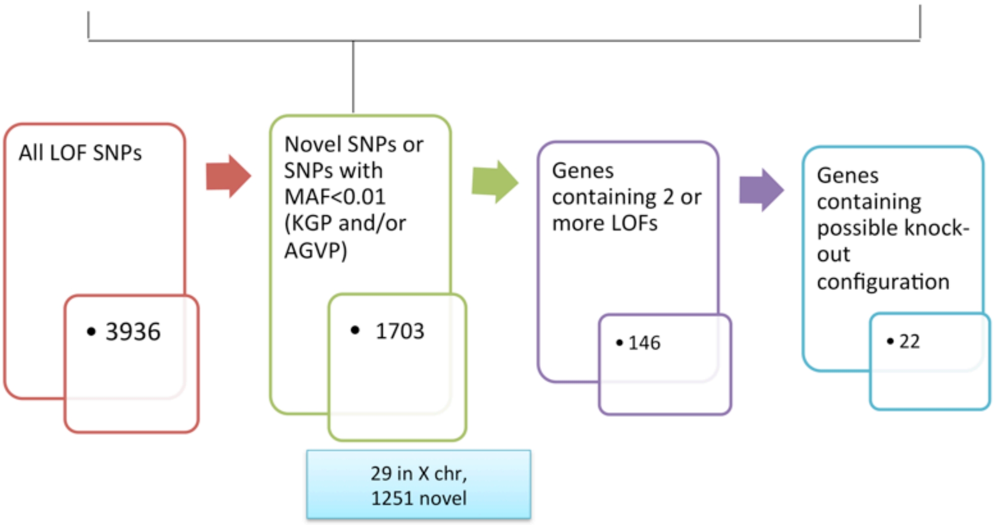

Supplementary Figure 3: Distribution of novel SNVs (a) Number of novel SNVs identified in individuals from the 4 populations. (b) The distribution of novel SNVs across the genome was found to be non-random with many regions showing extremely high enrichment. (c) The novel exonic SNVs were distributed more homogeneously with only a single region showing statistically significantly high enrichment. The black lines in (b) and (c) show Bonferroni corrected hypergeometric-p-value threshold for statistically significant enrichment.

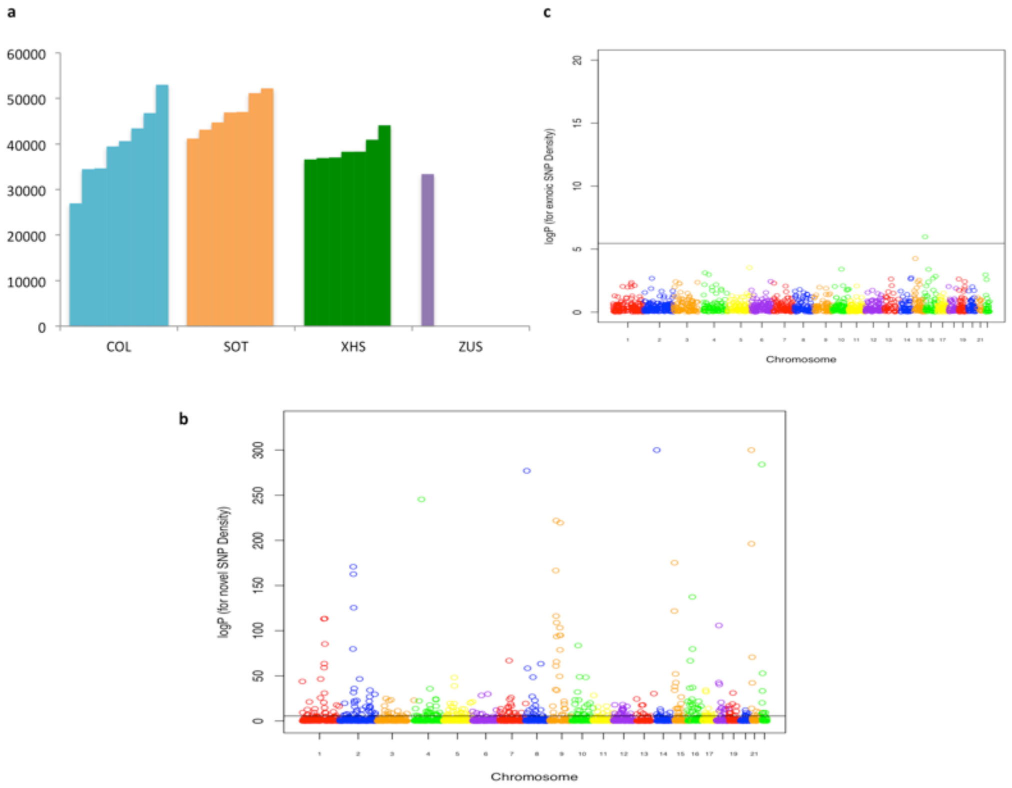

Supplementary Figure 4: The global principal component analysis. (a) PC2 and PC3. The SSMP have been removed for clarity as they cluster well away from the other data in this figure. (b) PC3 and PC4. The variation that each PC explains is shown on each axis.

a.

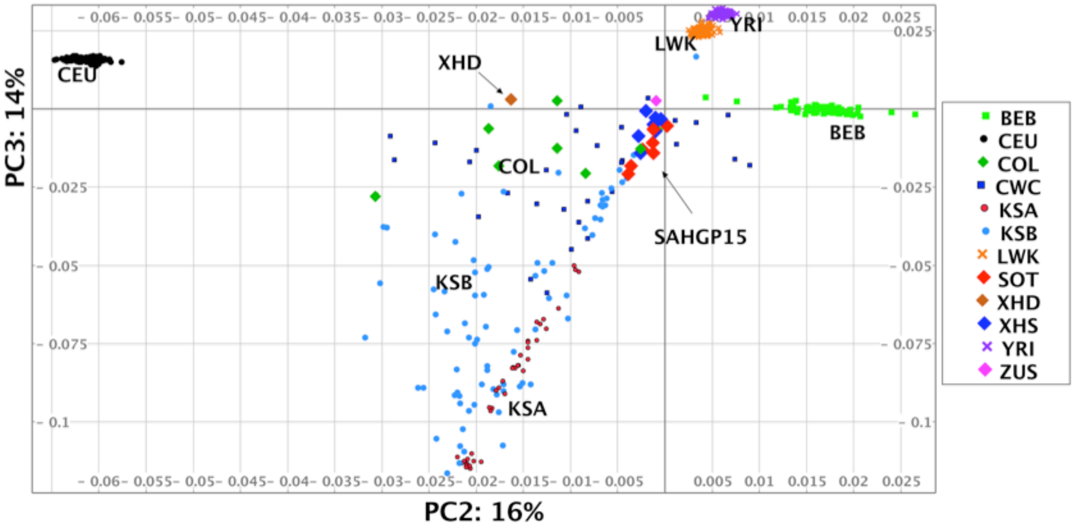

b)

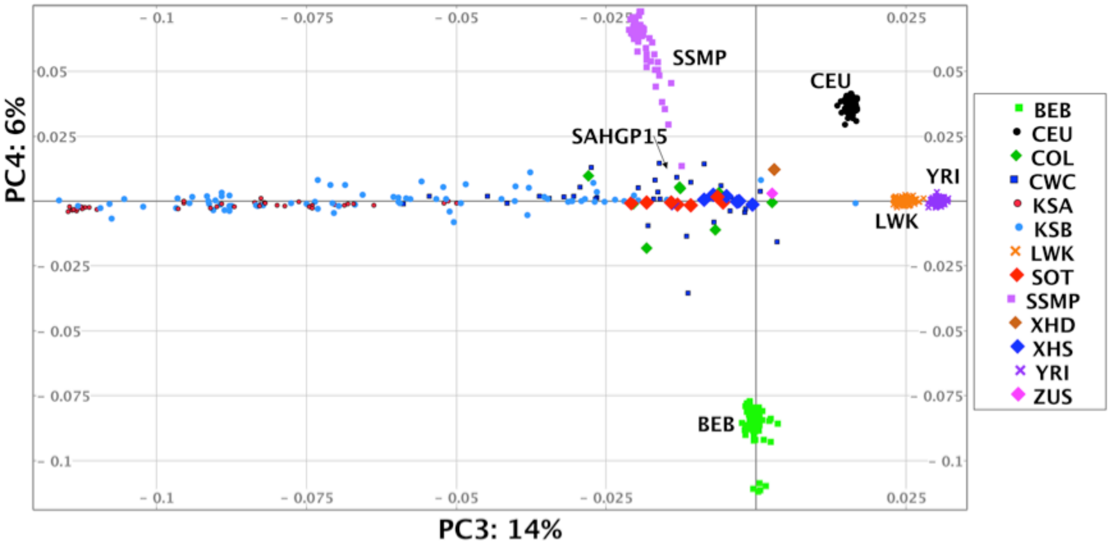

Supplementary Figure 5: **Comparison of ancestry proportions in SAHGP and related populations** (based on ADMIXTURE analyses) (a) in the two Coloured groups at *K*=10 (COL is based on the SAHGP data and COS is based on the Coloured population from Schlebusch *et al*. 2012). Only the ancestries showing discernible contributions are shown. In both the Coloured populations, the BEB appears to be the better South Asian proxy population in comparison to GIH. SSMP is the better East or Southeast Asian proxy population in comparison to CHB. Moreover, based on these novel proxies (BEB and SSMP, which were not included in previous studies) the South Asian contribution seems greater than that of the Southeast Asians. (b) The 3 southeastern Bantu-speaking (SEB) groups, the SOT and XHS from SAHGP and ZUL from the AGVP WGS dataset, are shown at *K*=7. The 4 ancestries demonstrating high contributions are shown (YRI, Khoisan (KS), SWB (South Western Bantu-speakers), SEB). The SOT show relatively higher KS ancestry in comparison to XHS and ZUL. (c) The relative ancestral population proportions are shown at *K*=7, the South African populations are highlighted. (d) Comparison of ancestry proportions in the northern (KSA), southern Khoesan group (KSB), SOT and XHS. The 3 ancestries showing high contributions are shown. The population labels in the headings of the columns in (c) and the legend in (d) represent the proxy ancestral groups – for example "SEB" should be read as "ancestral population most closely associated with SEB". The details for the population codes are listed in **Supplementary Table 10**.

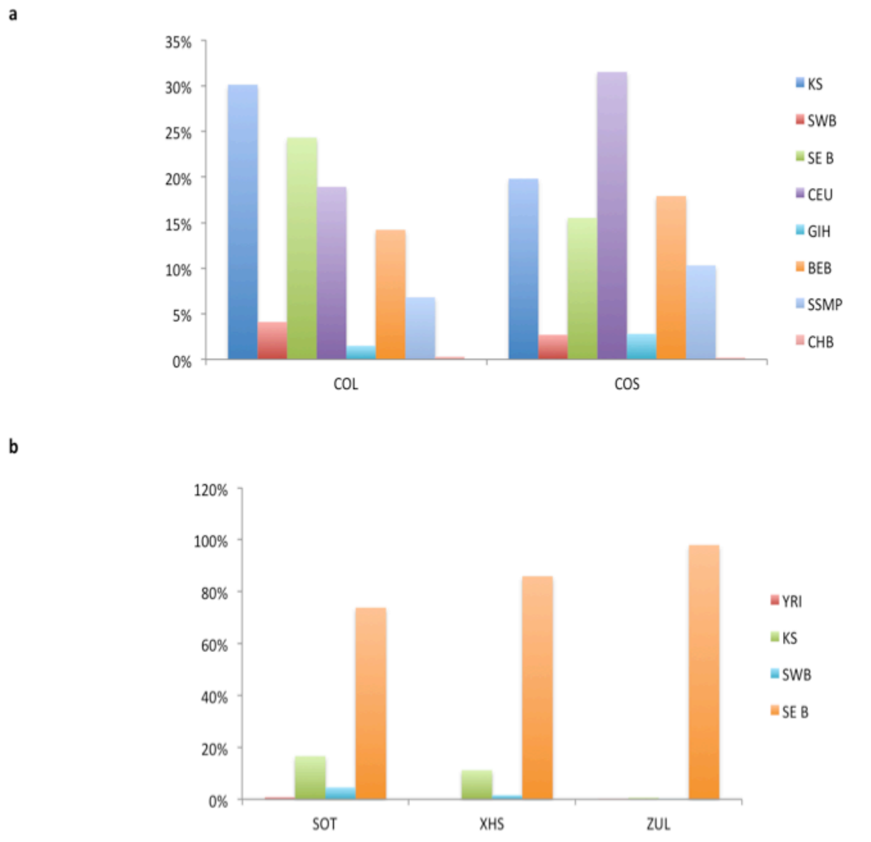

c

Pop	CEU	KS	SSMP	YRI	SEB	LWK	BEB
**BEB**	0.007	0.000	0.012	0.000	0.000	0.000	**0.981**
**COL**	0.238	0.227	0.063	0.019	0.277	0.037	0.140
**COS**	0.352	0.165	0.084	0.008	0.137	0.043	0.212
**CEU**	**1.000**	0.000	0.000	0.000	0.000	0.000	0.000
**KS**	0.060	**0.679**	0.003	0.073	0.104	0.065	0.016
**LWK**	0.000	0.013	0.000	0.029	0.019	**0.938**	0.000
**SEB**	0.006	0.062	0.000	0.055	**0.831**	0.042	0.004
**SOT**	0.000	0.161	0.000	0.019	**0.811**	0.008	0.000
**SSMP**	0.012	0.001	**0.919**	0.001	0.001	0.001	0.065
**SWB**	0.067	0.068	0.000	0.435	0.164	0.266	0.000
**XHD**	0.273	0.046	0.000	0.000	**0.681**	0.000	0.000
**XHS**	0.001	0.102	0.001	0.003	**0.893**	0.000	0.000
**YRI**	0.000	0.000	0.000	**0.969**	0.006	0.024	0.000
**ZUS**	0.018	0.039	0.000	0.017	**0.900**	0.027	0.000

d

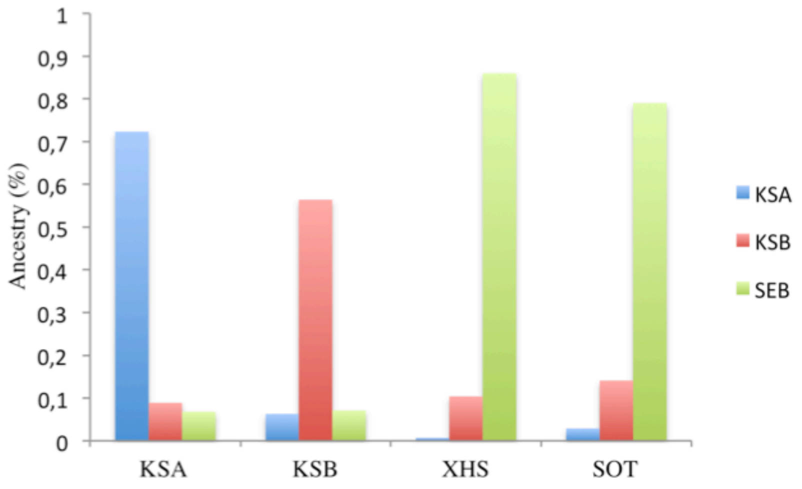

Supplementary Figure 6: **Principal component analysis showing only selected African populations.** The PCA was performed with data from all the selected populations but we only show some for clarity. (a) PC1 and PC 2 (b) also shows PC1 and PC2 but with participants from a study by May et al. 2013 consisting of residents from Soweto, Johannesburg (SEB3). Note that although the Johannesburg area is in a traditional Sotho-speaking area, the Soweto population is heterogeneous. Soweto has been urbanized for several generations and has drawn residents from across southern Africa. The ZUS individual was from Soweto. This figure shows the problem of language as a proxy for genetic background in an urbanized area. PC4 and PC5 (c), PC5 and PC6 (d), and PCA 1 and PCA 2 (e) show the ZUS individual in relation to the 7 SOT, 7 XHS and the AGVP ZUL individuals. The ZUS individual does not cluster with the AGVP ZUL individuals (these individuals were recruited from Kwa-Zulu Natal, a traditional Zulu-speaking region with less ethnic admixture). The details for the population codes are listed in **Supplementary Table 10**.

a.

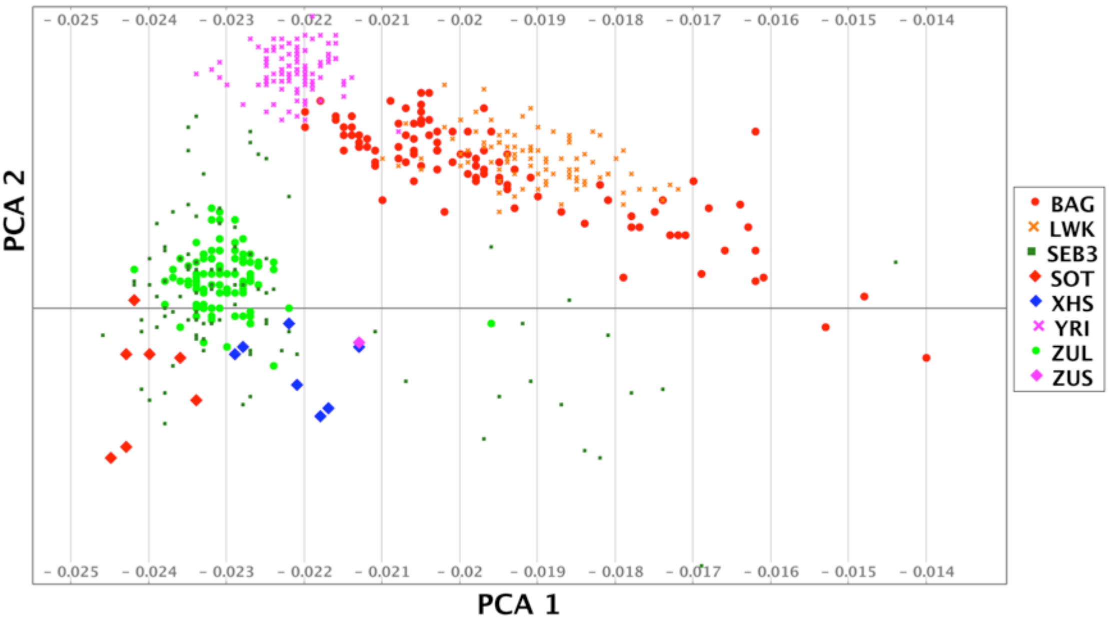

b

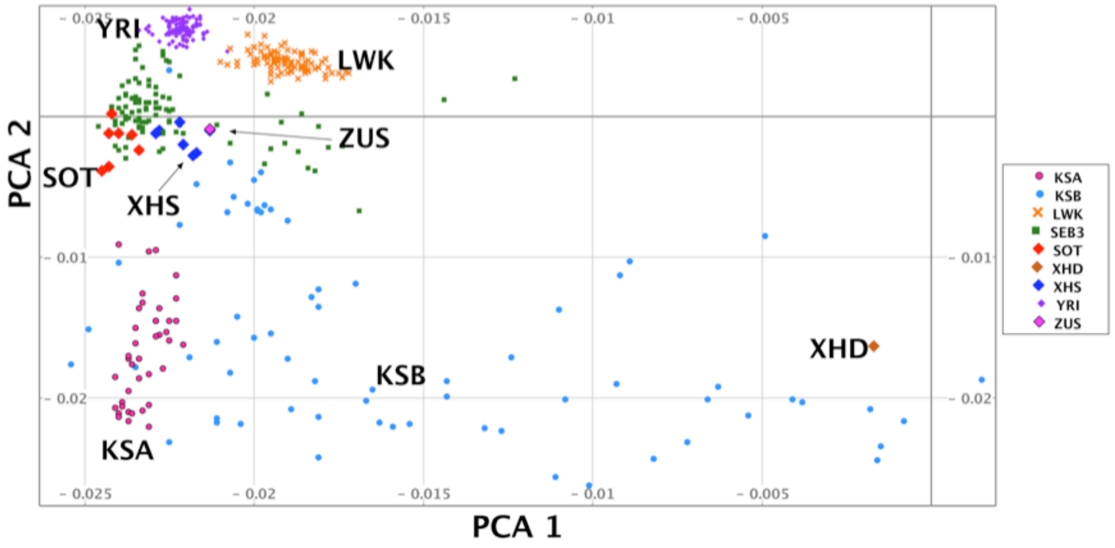
YRI
LWK
ZUS
SOT
XHS
KSB
XHD
KSA
PCA 2
PCA 1
KSA
KSB
LWK
SEB3
SOT
XHD
XHS
YRI
ZUS

c.

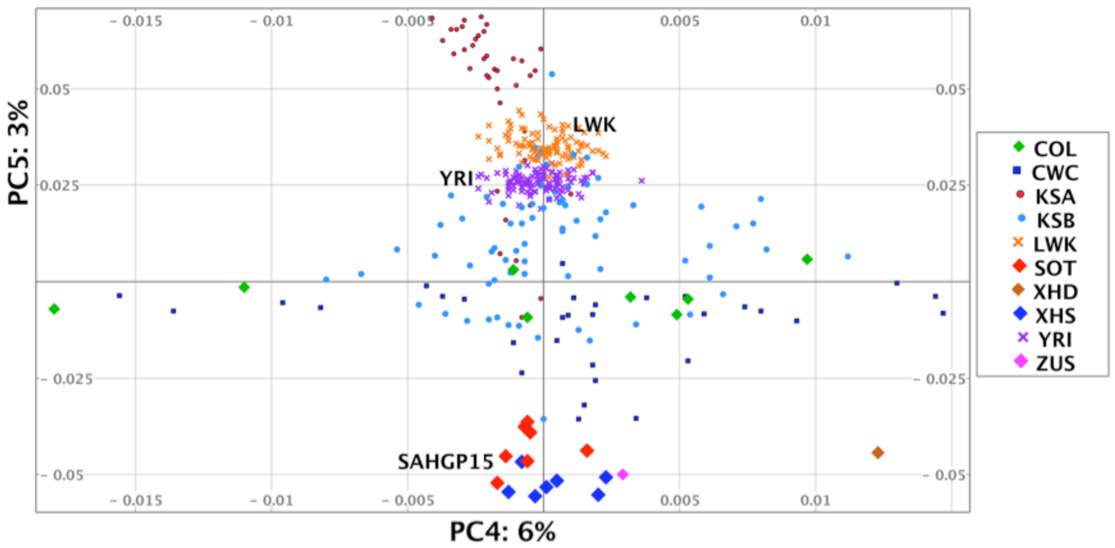
LWK
YRI
SAHGP15
PC5: 3%
PC4: 6%
COL
CWC
KSA
KSB
LWK
SOT
XHD
XHS
YRI
ZUS

d.

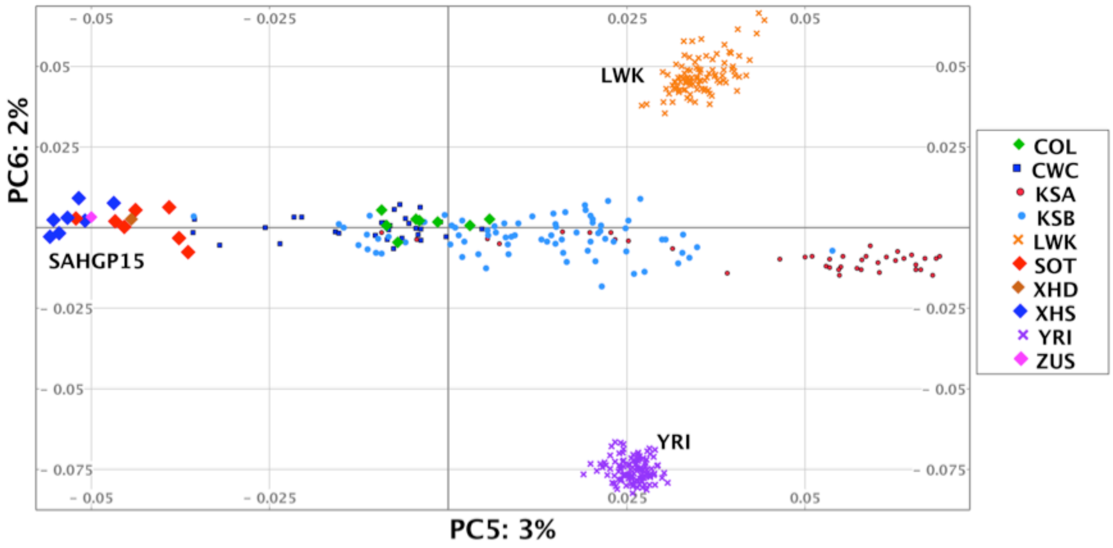

PC6: 2%
PC5: 3%
LWK
YRI
SAHGP15
COL
CWC
KSA
KSB
LWK
SOT
XHD
XHS
YRI
ZUS

e.

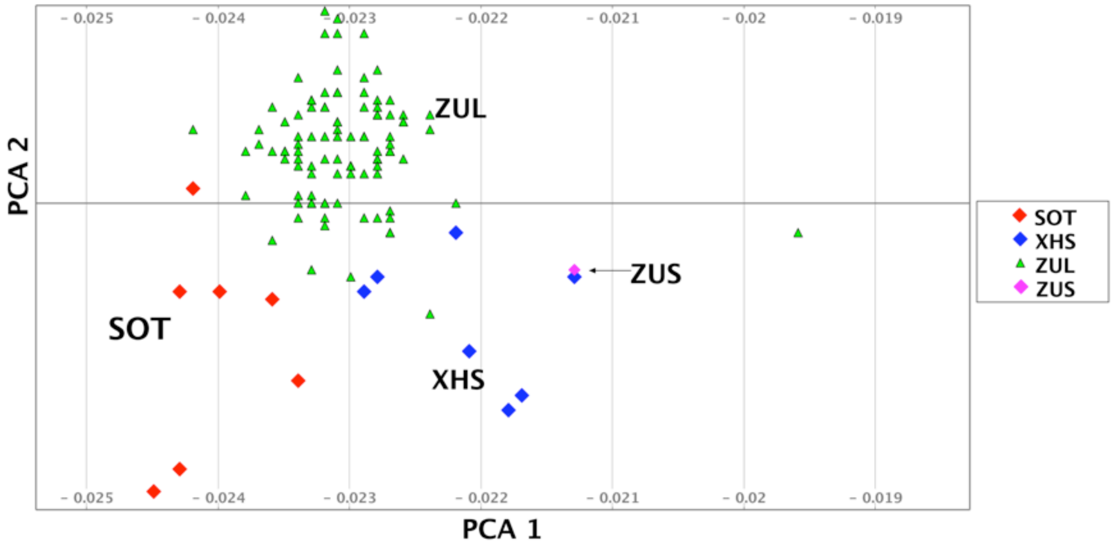

PCA 2
PCA 1
ZUL
ZUS
SOT
XHS
SOT
XHS
ZUL
ZUS

Supplementary Figure 7: Population structure of the SAHGP data for different values of *K*. (a) To the left of the line, each of the SAHGP individuals is shown, one person per column. To the right of the line, we summarize the populations showing the *average* of all members of that group. So, for example, it can be seen that the CEU individuals are on average close to 100% homogenous whereas for *K*=7, 61% of the KSB group ancestry is represented by the cyan colour. (b) Elaborates on 7a showing each person as a column for all populations. The SAHGP data is at the extreme left. The ZUS and XHD are in the data but at this level of resolution cannot be labelled. (c) Estimating the best *K* value to use for structure. The figure shows the cross-validation scores that ADMIXTURE estimates. *K*=7 has the lowest score. Lower values for *K* also have obvious anomalies. *K*=8 or 9 may also be reasonable values to use. The details for the population codes are listed in **Supplementary Table 10**.

a

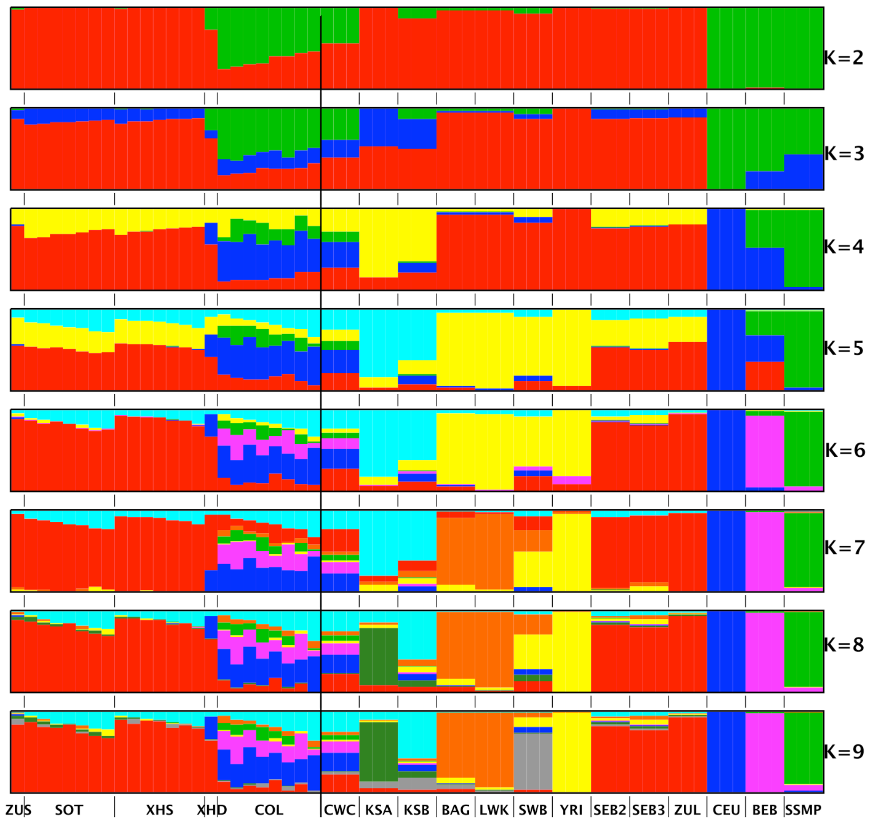

b

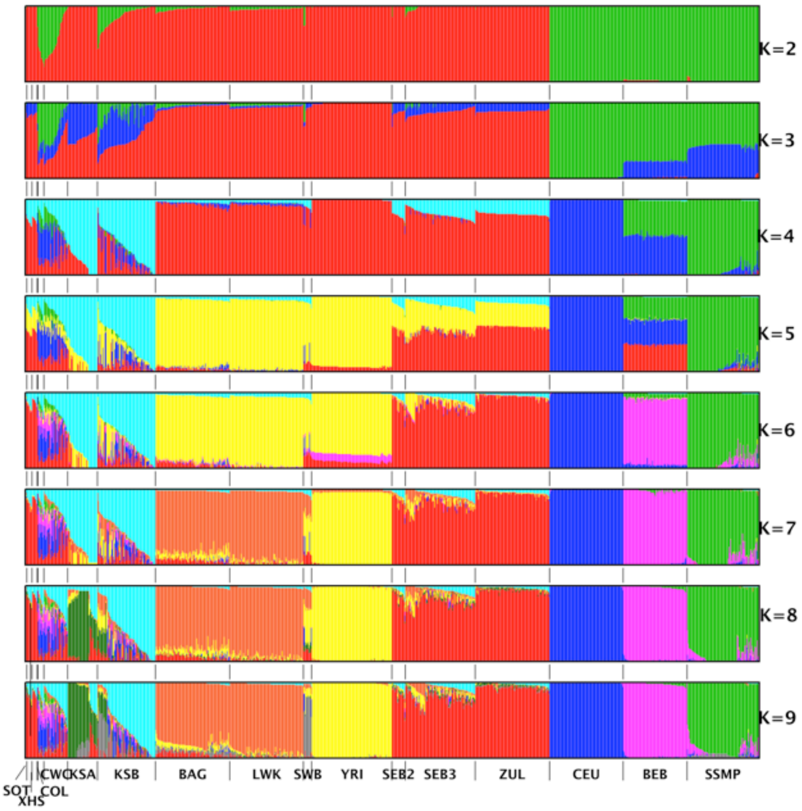
K=2
K=3
K=4
K=5
K=6
K=7
K=8
K=9
SOT
XHS
CWC
COL
KSA
KSB
BAG
LWK
SWB
YRI
SEB2
SEB3
ZUL
CEU
BEB
SSMP

c

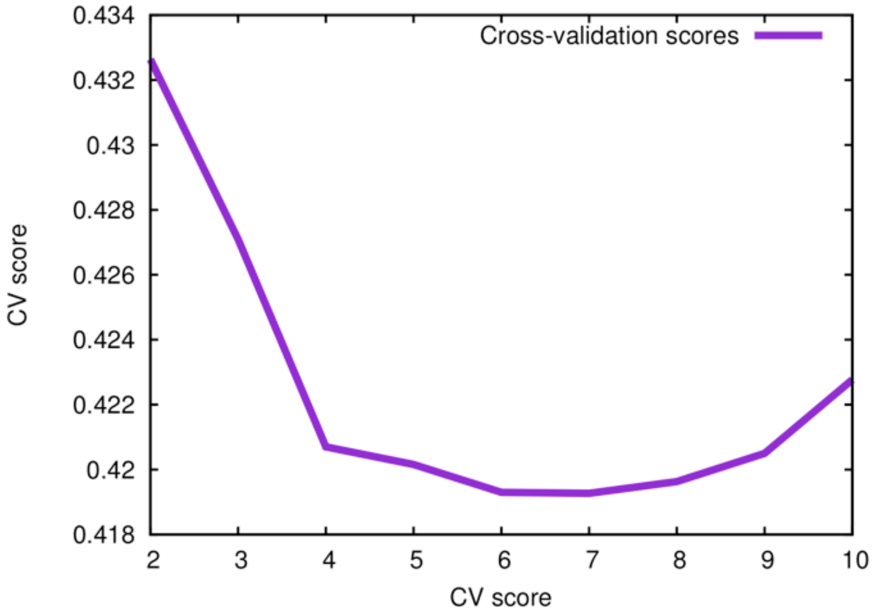
Cross-validation scores
0.434
0.432
0.43
0.428
0.426
0.424
0.422
0.42
0.418
CV score
2
3
4
5
6
7
8
9
10
CV score

Supplementary Figure 8: Manhattan plot showing regional F_{ST} score variation between SOT and XHS (based on 25 Kb sliding windows). Y-axis shows average F_{ST} values for each sliding window.

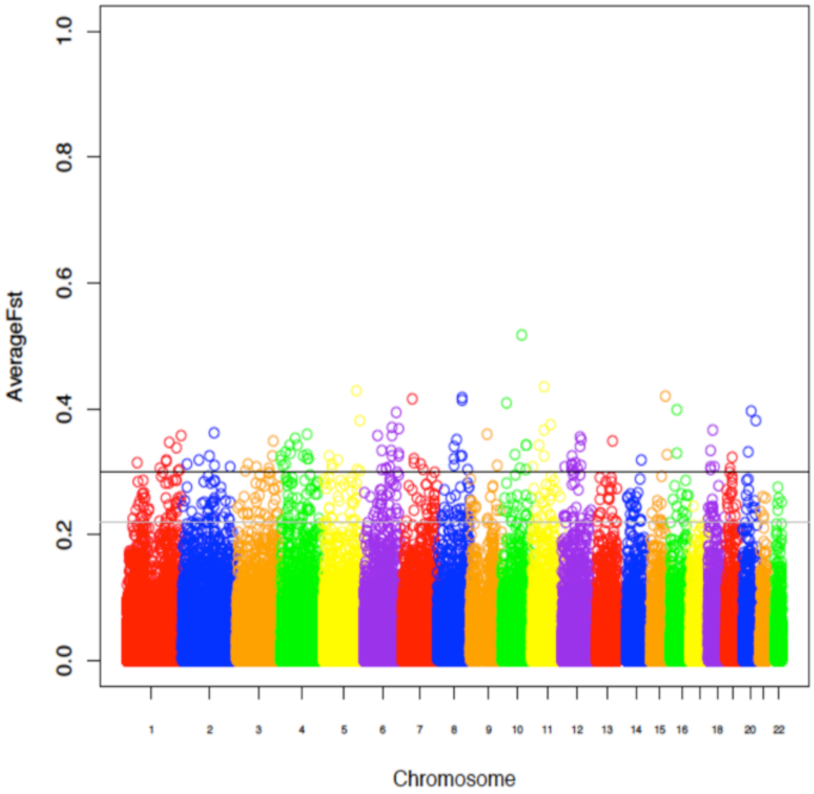

Supplementary Figure 9: Analysis of SNVs shared between SAHGP and the KGP populations irrespective of minor allele frequency. (a) The frequency of SNVs shared between various KGP populations and the 15 SEB speakers (the SNVs occurring in the SEB and only one of the KGP populations were considered). (b) The frequency of SNVs shared between various KGP populations and COL individuals (the SNVs occurring in the COL and only one of the KGP populations were considered). The East African populations are shown in red, the West Africans and admixed African in green and other populations are shown in blue. (c) Continent/Super-population specific SNVs in KGP that were detected in the 15 SEB (shown in blue) and COL (shown in red). The details for the population codes are listed in **Supplementary Table 10**.

a

b

c.

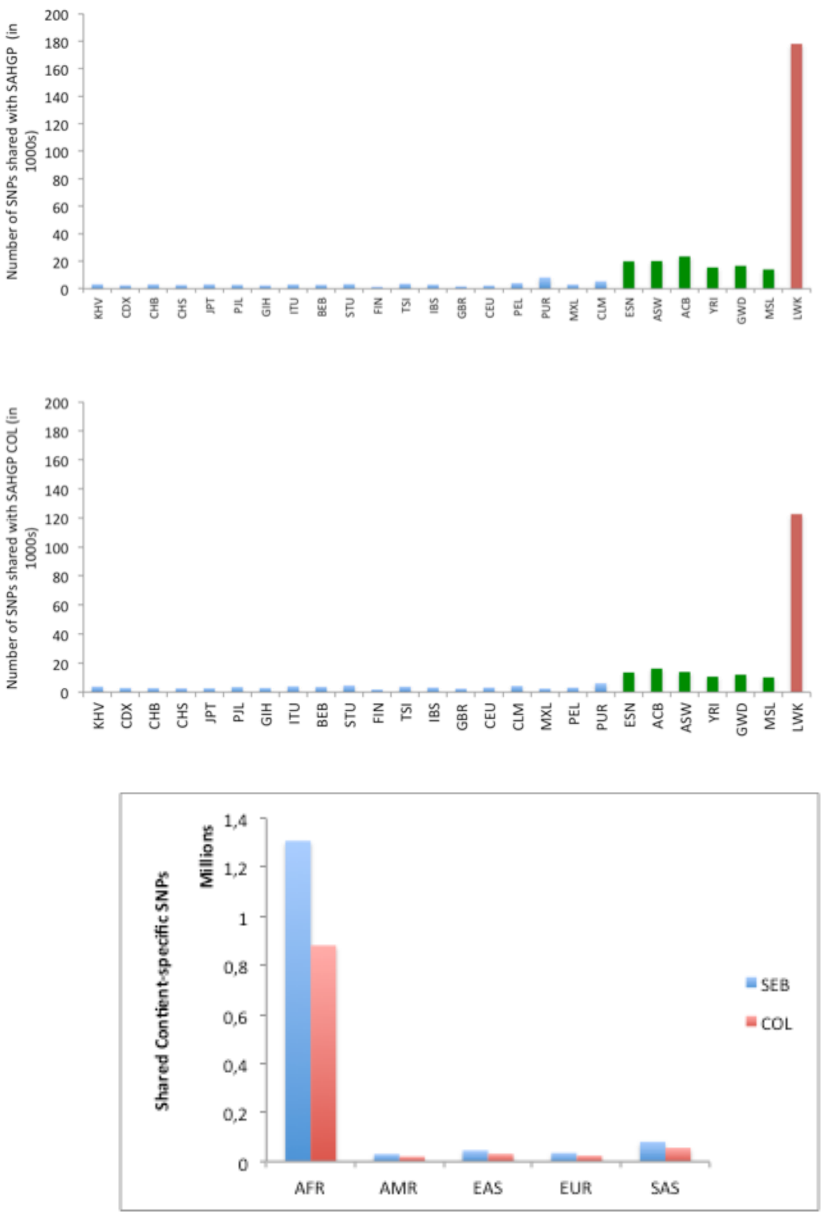

Supplementary Figure 10: F_{ST} based estimates of genetic distance between the SAHGP and other African populations. (a) F_{ST} estimates for distance between the three SAHGP populations Sotho (SOT), Xhosa (XHS) and Coloured (COL) and various Khoesan groups based on the non-Niger-Congo ancestry masked dataset. The figure shows that trend observed in **Figure 3d** was not altered significantly due to maksing. A suffix "_m" has been added to each population name to indicate that the results are based on an ancestry-masked dataset. (b) Genetic distances between SOT, XHS and other Bantu-speaking populations. The population details and data sources are detailed in **Supplementary Table 10**.

a.

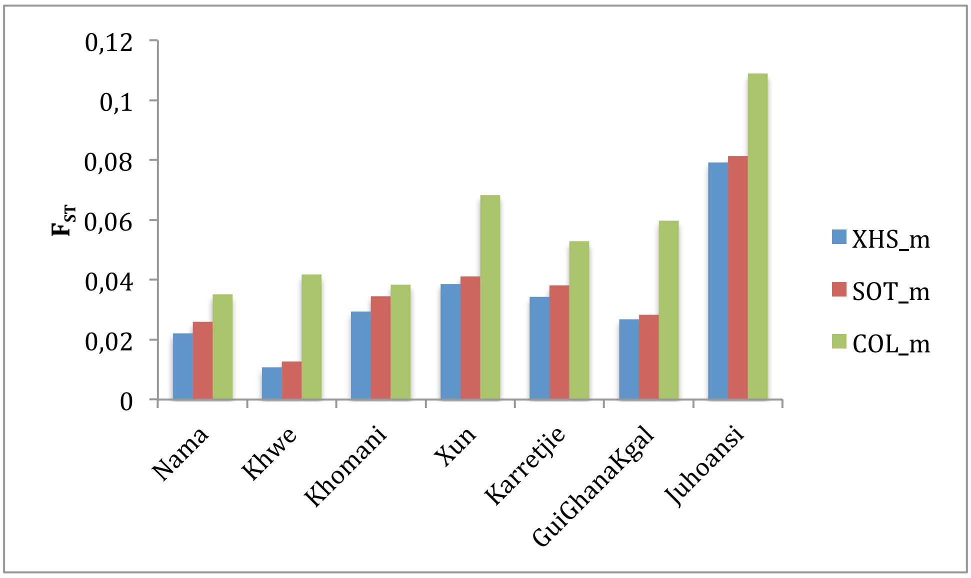

b.

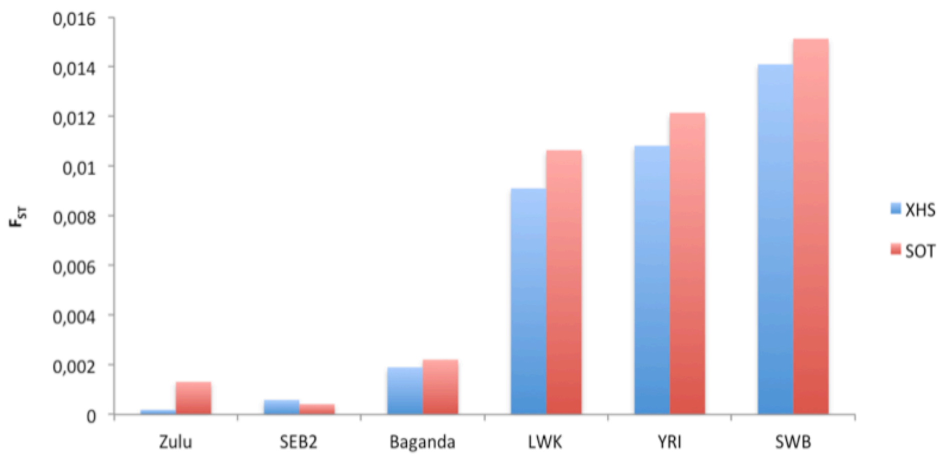

Supplementary Figure 11: Total ROH length variation within African and non-African populations (a) Map showing the distribution of African population groups or super-populations used in this analysis. The map was generated using SimpleMappr (http://www.simplemappr.net/). (b) Violin plots summarizing total ROH length in various population groups. The colours of the population groups in the map and the violin plots are matched. The coloured boxes in (a) correspond to the violin plot colours in (b). (c) p-values based on Mann-Whitney test (P<0.00001 highlighted in red).

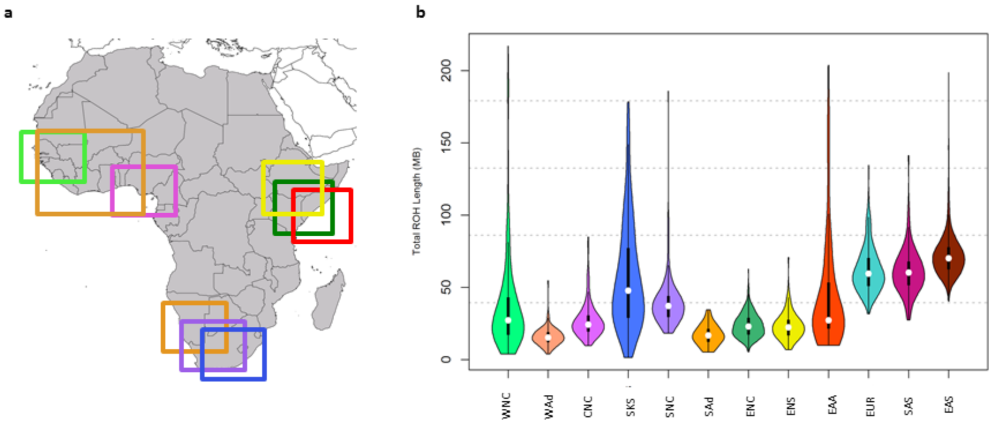

c

POP	WNC	WAd	CNC	SKS	SNC	SAd	ENC	ENS	EAA	EUR	SAS	EAS
WNC	1,00E+00	8,01E-22	4,94E-02	2,14E-13	2,21E-10	8,49E-09	2,82E-06	6,25E-04	2,06E-02	1,73E-63	2,36E-24	9,42E-77
WAd	8,01E-22	9,99E-01	3,68E-29	1,09E-34	4,72E-49	2,59E-01	5,80E-24	1,99E-12	5,42E-26	4,99E-64	6,25E-36	3,18E-64
CNC	4,94E-02	3,68E-29	1,00E+00	3,56E-25	1,96E-32	2,96E-10	1,35E-03	6,94E-03	7,57E-05	3,66E-106	1,94E-41	6,79E-113
SKS	2,14E-13	1,09E-34	3,56E-25	9,99E-01	1,93E-05	3,04E-18	6,96E-36	2,01E-20	1,56E-05	3,43E-06	2,15E-03	2,98E-14
SNC	2,21E-10	4,72E-49	1,96E-32	1,93E-05	1,00E+00	1,70E-23	2,24E-54	5,38E-26	8,22E-04	5,35E-68	2,92E-28	8,41E-86
SAd	8,49E-09	2,59E-01	2,96E-10	3,04E-18	1,70E-23	9,97E-01	1,47E-07	3,36E-05	1,90E-12	3,25E-30	2,45E-22	3,88E-30
ENC	2,82E-06	5,80E-24	1,35E-03	6,96E-36	2,24E-54	1,47E-07	1,00E+00	4,37E-01	1,91E-09	1,23E-152	5,16E-51	6,10E-153
ENS	6,25E-04	1,99E-12	6,94E-03	2,01E-20	5,38E-26	3,36E-05	4,37E-01	9,99E-01	4,77E-07	3,61E-53	2,92E-31	2,96E-54
EAA	2,06E-02	5,42E-26	7,57E-05	1,56E-05	8,22E-04	1,90E-12	1,91E-09	4,77E-07	9,99E-01	3,10E-20	1,11E-11	2,01E-26
EUR	1,73E-63	4,99E-64	3,66E-106	3,43E-06	5,35E-68	3,25E-30	1,23E-152	3,61E-53	3,10E-20	1,00E+00	8,11E-01	1,15E-29
SAS	2,36E-24	6,25E-36	1,94E-41	2,15E-03	2,92E-28	2,45E-22	5,16E-51	2,92E-31	1,11E-11	8,11E-01	9,99E-01	6,11E-13
EAS	9,42E-77	3,18E-64	6,79E-113	2,98E-14	8,41E-86	3,88E-30	6,10E-153	2,96E-54	2,01E-26	1,15E-29	6,11E-13	1,00E+00

Supplementary Tables

Supplementary Table 1: Sequencing statistics for the 24 samples. (a) Alignment metrics for the 24 samples

Population	Sample	Total Reads (Passed QC)	Duplicates	Mapped	Paired
COL	A	1544212526	32807632 (2.12%)	1441441080 (93.34%)	1412962086 (91.50%)
COL	B	1554671102	32127032 (2.07%)	1452569542 (93.43%)	1424468510 (91.63%)
COL	C	1581890206	33407004 (2.11%)	1479514713 (93.53%)	1450006260 (91.66%)
COL	D	1545665680	36065726 (2.33%)	1437182059 (92.98%)	1408479462 (91.12%)
COL	E	1578794344	43947664 (2.78%)	1468313554 (93.00%)	1437795842 (91.07%)
COL	F	1597786856	46259390 (2.90%)	1492257225 (93.40%)	1464241976 (91.64%)
COL	G	1565436298	42841136 (2.74%)	1459746562 (93.25%)	1431037710 (91.41%)
COL	H	1347238002	25276290 (1.88%)	1258959994 (93.45%)	1235277784 (91.69%)
ZUS	ZUS	1482018800	45881492 (3.10%)	1381255973 (93.20%)	1352249522 (91.24%)
SOT	A	1665874218	33564922 (2.01%)	1559297227 (93.60%)	1528252438 (91.74%)
SOT	B	1587770692	30950706 (1.95%)	1484344595 (93.49%)	1455535586 (91.67%)
SOT	C	1503836688	30059592 (2.00%)	1400330926 (93.12%)	1369812454 (91.09%)
SOT	D	1346754898	26401714 (1.96%)	1255146023 (93.20%)	1229157734 (91.27%)
SOT	E	1026031844	15224188 (1.48%)	961002109 (93.66%)	942519738 (91.86%)
SOT	F	1462170384	25507566 (1.74%)	1364327381 (93.31%)	1336389766 (91.40%)
SOT	G	1625322888	30177840 (1.86%)	1522313925 (93.66%)	1490727214 (91.72%)
XHS	A	1463087870	25958626 (1.77%)	1363792197 (93.21%)	1334044114 (91.18%)
XHS	B	1404194462	25461008 (1.81%)	1309595450 (93.26%)	1279263780 (91.10%)
XHS	C	1404569154	25917666 (1.85%)	1306584392 (93.02%)	1276063236 (90.85%)
XHS	D	1418667140	28935836 (2.04%)	1324834835 (93.39%)	1297251696 (91.44%)
XHS	E	1407021356	27267098 (1.94%)	1312743417 (93.30%)	1285772642 (91.38%)
XHS	F	1491021258	30995878 (2.08%)	1391299534 (93.31%)	1363425976 (91.44%)
XHS	G	1491562956	32573452 (2.18%)	1391617313 (93.30%)	1362853388 (91.37%)
XHS	H	1471866322	28777378 (1.96%)	1375200936 (93.43%)	1347633400 (91.56%)

(b) Coverage observed in the 24 samples

Population ID	Sample ID	Coverage
COL	A	49.62
	B	50.08
	C	51.02
	D	49.38
	E	50.29
	F	51.06
	G	50.05
	H	43.58
ZUS	ZUS	47.12
SOT	A	53.78
	B	51.2
	C	48.32
	D	43.32
	E	33.38
	F	47.25
	G	52.63
XHS	A	47.22
	B	45.25
	C	45.14
	D	45.69
	E	45.31
	F	47.93
	G	47.8
	H	47.47

Supplementary Table 2: SNV statistics per individual. (a) Number of SNVs called in the initial variant calling (SNVs) and the number of SNVs called by all three methods (Recalled SNVs).

Population ID	Sample ID	SNVs	Recalled SNVs
COL	A	3915139	3794641
	B	4084529	3962793
	C	3891282	3774923
	D	3849361	3732992
	E	3984479	3861296
	F	4093220	3970458
	G	4148078	4021907
	H	4029490	3908608
ZUS	ZUS	4363081	4246441
SOT	A	4472758	4351597
	B	4441572	4319446
	C	4411497	4290675
	D	4423145	4304039
	E	4383320	4266223
	F	4417550	4298337
	G	4463322	4344196
XHS	A	4365731	4243481
	B	4394688	4275127
	C	4400000	4278927
	D	4385230	4263928
	E	4378595	4260540
	F	4397950	4274731
	G	4385380	4262823
	H	4243861	4122619

(b) Basic statistics for recalled SNVs. The TiTv ratios for known and novel SNVs are shown separately.

Population	Sample	Heterozygous/Homozygous Ratio	dbSNP TiTv Ratio	Novel SNP TiTv Ratio	No. of Singletons
COL	A	1.905244	2.120055	1.993457	200547
	B	1.953055	2.122974	1.999439	231653
	C	1.920255	2.119414	2.008787	200978
	D	1.833636	2.123652	1.996636	209486
	E	1.903616	2.119658	2.023491	220691
	F	1.960642	2.118108	2.011759	221709
	G	1.998045	2.12601	2.019922	245757
	H	1.960748	2.117505	2.006286	220975
ZUS	ZUS	1.881792	2.12023	1.956072	230810
SOT	A	1.911439	2.122469	1.984833	256634
	B	1.884215	2.12366	1.970153	255116
	C	1.880955	2.126693	1.980289	243640
	D	1.911883	2.122814	1.934572	245470
	E	1.93983	2.123817	1.987142	251025
	F	1.87151	2.125214	1.967278	242795
	G	1.854771	2.123012	2.006098	250476
XHS	A	1.885242	2.123635	1.954718	230398
	B	1.899185	2.122778	1.98766	238909
	C	1.900806	2.12358	1.986435	243881
	D	1.911527	2.122263	1.988505	233056
	E	1.862195	2.122775	1.969349	230666
	F	1.921636	2.121881	1.975089	236769
	G	1.936089	2.12572	2.012286	236492

Supplementary Table 3: Indels and CNVs – average across the groups.

a. Indels observed in 24 individuals from the three populations

Sample	COL	SOT	XHS	ZUS
A	698604	764205	774040	780394
B	719904	763834	796285	
C	686103	765052	786192	
D	679573	766096	784312	
E	721694	772035	779611	
F	739572	771382	745236	
G	745618	744892	794163	
H	711170	-	807985	
AVERAGE	712780	763928	783478	780394

b. CNVs observed in 24 individuals from the three populations.

Sample	COL	SOT	XHS	ZUS
A	51	71	73	77
B	68	79	85	
C	76	64	78	
D	60	91	66	
E	55	91	83	
F	55	79	77	
G	55	82	59	
H	53	-	64	
AVERAGE	59	80	73	77

Supplementary Table 4: Genic annotation and potential functional variation of SNVs. Annotation was performed with the ANNOVAR (2015Mar22) software and database (accessed on 22nd March 2015). A variant type count is only reported when 2 or more samples in a population had the variant. The single ZUS individual was added to SOT for this comparison.

	COL	SOT	XHO
**Variant type**			
Downstream	328905	362839	310062
Exonic	189776	209019	178581
Exonic and splicing	196	173	146
Intergenic	14633927	15927468	13620631
Intronic	10542374	11599791	9926636
ncRNA_exonic	307117	338015	289214
ncRNA_exonic and splicing	248	275	214
ncRNA_intronic	3410568	3744822	3197547
ncRNA_splicing	1427	1537	1351
Splicing	1273	1322	1174
Upstream	308078	339093	288677
Upstream and downstream	21033	20488	17509
UTR3	234439	260215	223240
UTR5	70685	77696	66703
UTR5 and UTR3	1023	960	902
**Exonic variant type**			
Stopgain	853	985	793
Stoploss	392	406	349
Nonsynonymous SNV	89295	98450	83906
Synonymous SNV	96235	105830	90714
Unknown	3108	3430	2888

Supplementary Table 5: Genic annotation and potential functional variation of indels. Annotation was performed with the ANNOVAR software and database (accessed on 22nd March 2015). A variant type count is only reported when 2 or more samples in a population had the variant. The single ZUS individual was added to SOT for this comparison.

	COL	SOT	XHO
**Variant type**			
Downstream	78773	77093	65585
Exonic	6355	5883	4965
Exonic and splicing	189	197	167
Intergenic	2877558	2801351	2381826
Intronic	2280262	2216912	1875344
ncRNA_exonic	42012	41173	34992
ncRNA_exonic and splicing	169	172	158
ncRNA_intronic	714873	696843	589335
ncRNA_splicing	414	342	342
Splicing	565	610	540
Upstream	68236	66643	56063
Upstream and downstream	4765	4496	3997
UTR3	58695	57075	48659
UTR5	12066	11742	9784
UTR5 and UTR3	162	153	128
**Exonic variant type**			
Stopgain	73	77	67
Stoploss	9	8	4
Nonsynonymous SNV	0	0	0
Synonymous SNV	0	0	0
Unknown	386	401	34

Supplementary Table 6: Potential knockouts detected in the SAHGP individuals. (a) The genotypes in each individual from all 4 populations are shown. Homozygous individuals are shown by "-". Potential knockout configurations are shown in bold.

SNP (Chr_Pos)	COL_A	COL_A	COL_B	COL_B	COL_C	COL_C	COL_D	COL_D	COL_E	COL_E	COL_F	COL_F	COL_G	COL_G	COL_H	COL_H
5_668574	-	-	-	-	-	-	**CT**	**C**	-	-	-	-	**CT**	**C**	-	-
5_668654	-	-	-	-	-	-	**T**	**TCAGA**	-	-	-	-	**T**	**TCAGA**	-	-
7_105641910	-	-	-	-	-	-	-	-	-	-	-	-	-	-	-	-
7_105668924	-	-	-	-	-	-	-	-	-	-	-	-	-	-	-	-
8_22432388	-	-	-	-	-	-	-	-	-	-	-	-	**A**	**AC**	-	-
8_22432396	-	-	-	-	-	-	-	-	-	-	-	-	**T**	**C**	-	-
10_51748681	-	-	**A**	**AC**	-	-	-	-	-	-	-	-	A	AC	-	-
10_51768674	-	-	**C**	**CAA**	-	-	-	-	**C**	**CAA**	-	-	-	-	-	-
14_60448779	-	-	-	-	-	-	-	-	-	-	-	-	-	-	**A**	**G**
14_60474859	-	-	-	-	-	-	-	-	-	-	-	-	-	-	**A**	**G**
19_42747163	-	-	-	-	**T**	**TTGCAGGTG**	-	-	-	-	-	-	-	-	-	-
19_42747179	-	-	-	-	**A**	**AT**	-	-	-	-	-	-	-	-	-	-
19_54803664	**A**	**C**	-	-	-	-	-	-	-	-	-	-	-	-	-	-
19_54803979	**G**	**C**	-	-	-	-	-	-	-	-	-	-	-	-	-	-
20_61588315	**T**	**G**	-	-	-	-	-	-	-	-	-	-	-	-	-	-
20_61588316	**C**	**T**	-	-	-	-	-	-	-	-	-	-	-	-	-	-
**SNP (Chr_Pos)**	**XHS_A**	**XHS_A**	**XHS_B**	**XHS_B**	**XHS_C**	**XHS_C**	**XHS_D**	**XHS_D**	**XHS_E**	**XHS_E**	**XHS_F**	**XHS_F**	**XHS_G**	**XHS_G**	**ZUS**	**ZUS**
3_146177635	-	-	-	-	-	-	-	-	-	-	-	-	**C**	**CTGACT**	**C**	**CT**
3_146179745	TA	T	-	-	-	-	-	-	-	-	-	-	**TA**	**T**	**T**	**TG**
8_22432388	-	-	-	-	A	AC	-	-	-	-	**A**	**AC**	-	-		
8_22432396	-	-	-	-	T	C	-	-	-	-	**T**	**C**	-	-		
10_51748528	-	-	**AC**	**A**	-	-	-	-	AC	A	-	-	-	-		
10_51768674	-	-	**C**	**CAA**	-	-	-	-	-	-	-	-	-	-		
11_18727647	-	-	-	-	-	-	**C**	**CCA**	-	-	-	-	-	-		
11_18728743	-	-	-	-	-	-	**T**	**TG**	-	-	-	-	-	-		
12_131514221	-	-	**A**	**AT**	-	-	-	-	-	-	-	-	-	-		
12_131514265	-	-	**TA**	**T**	-	-	-	-	-	-	-	-	-	-		
12_131514761	-	-	**AC**	**A**	-	-	-	-	-	-	-	-	-	-		
13_49775314	-	-	**C**	**CAT**	-	-	-	-	-	-	-	-	-	-		
13_49775366	-	-	**G**	**A**	-	-	-	-	-	-	-	-	-	-		

19_13899019	-	-	-	-	-	-	-	-	-	-	**T**	**C**	-	-		
19_13899040	-	-	-	-	-	-	-	-	-	-	**T**	**TC**	-	-		
19_53454007	**A**	**AAG**	-	-	-	-	-	-	-	-	-	-	-	-		
19_53454370	**A**	**G**	-	-	-	-	-	-	-	-	-	-	-	-		
**SNP (Chr_Pos)**	**SOT_A**	**SOT_A**	**SOT_B**	**SOT_B**	**SOT_C**	**SOT_C**	**SOT_D**	**SOT_D**	**SOT_E**	**SOT_E**	**SOT_F**	**SOT_F**	**SOT_G**	**SOT_G**	**#**	**#**
4_109681449	-	-	-	-	-	-	-	-	-	-	-	-	-	-		
4_109681452	-	-	-	-	-	-	-	-	-	-	-	-	-	-		
4_69796262	-	-	-	-	-	-	**G**	**A**	-	-	-	-	-	-		
4_69817185	-	-	-	-	-	-	**C**	**CA**	-	-	-	-	-	-		
5_668574	-	-	-	-	-	-	-	-	-	-	CT	C	-	-		
7_105641910	-	-	-	-	-	-	-	-	-	-	**T**	**C**	-	-		
7_105668924	A	G	-	-	-	-	A	G	-	-	**A**	**G**	-	-		
10_51748681	-	-	A	AC	A	AC	-	-	-	-	-	-	-	-		
12_131514264	-	-	-	-	-	-	**G**	**GT**	-	-	-	-	-	-		
12_131514265	-	-	-	-	-	-	-	-	-	-	-	-	-	-		
12_131514761	-	-	-	-	-	-	**AC**	**A**	-	-	AC	A	-	-		
19_13899019	-	-	-	-	-	-	-	-	-	-	T	C	-	-		

(b) Details of the Loss of function SNVs included in (a)

**SNP (Chr_Pos)**	**Type**	**Gene**	**Description**
3_146177635	frameshift_deletion	*PLSCR2*	phospholipid scramblase 2
3_146179745	splicing	*PLSCR2*	phospholipid scramblase 2
4_109681449	frameshift_deletion	*ETNPPL*	ethanolamine-phosphate phospho-lyase
4_109681452	stopgain	*ETNPPL*	ethanolamine-phosphate phospho-lyase
4_69796262	splicing	*UGT2A3*	UDP glucuronosyltransferase 2 family, polypeptide A3
4_69817185	frameshift_deletion	*UGT2A3*	UDP glucuronosyltransferase 2 family, polypeptide A3
5_668574	frameshift_insertion	*AC026740.1*	Uncharacterized protein
5_668654	frameshift_deletion	*AC026740.1*	Uncharacterized protein
7_105641910	stopgain	*CDHR3*	cadherin-related family member 3
7_105668924	splicing	*CDHR3*	cadherin-related family member 3
8_22432388	frameshift_deletion	*SORBS3*	sorbin and SH3 domain containing 3
8_22432396	stopgain	*SORBS3*	sorbin and SH3 domain containing 3
10_51748528	frameshift_insertion	*AGAP6*	ArfGAP with GTPase domain, ankyrin repeat and PH domain 6
10_51748681	frameshift_deletion	*AGAP6*	ArfGAP with GTPase domain, ankyrin repeat and PH domain 6
10_51768674	frameshift_deletion	*AGAP6*	ArfGAP with GTPase domain, ankyrin repeat and PH domain 6
11_18727647	frameshift_deletion	*IGSF22*	immunoglobulin superfamily, member 22
11_18728743	frameshift_deletion	*IGSF22*	immunoglobulin superfamily, member 22

12_131514221	frameshift_deletion	*AC078925.1*	Uncharacterized protein
12_131514264	frameshift_deletion	*AC078925.1*	Uncharacterized protein
12_131514265	frameshift_insertion	*AC078925.1*	Uncharacterized protein
12_131514761	frameshift_insertion	*AC078925.1*	Uncharacterized protein
13_49775314	frameshift_deletion	*FNDC3A*	fibronectin type III domain containing 3A
13_49775366	splicing	*FNDC3A*	fibronectin type III domain containing 3A
14_60448779	splicing	*LRRC9*	leucine rich repeat containing 9
14_60474859	stopgain	*LRRC9*	leucine rich repeat containing 9
19_13899019	splicing	*AC008686.1*	Uncharacterized protein
19_13899040	frameshift_deletion	*AC008686.1*	Uncharacterized protein
19_42747163	frameshift_deletion	*AC006486.1*	
19_42747179	frameshift_deletion	*AC006486.1*	
19_53454007	frameshift_deletion	*ZNF816*	zinc finger protein 816
19_53454370	stopgain	*ZNF816*	zinc finger protein 816
19_54803664	stopgain	*LILRA3*	leukocyte immunoglobulin-like receptor, subfamily A (without TM domain), member
19_54803979	splicing	*LILRA3*	leukocyte immunoglobulin-like receptor, subfamily A (without TM domain), member 3
20_61588315	splicing	*SLC17A9*	solute carrier family 17 (vesicular nucleotide transporter), member 9
20_61588316	splicing	*SLC17A9*	solute carrier family 17 (vesicular nucleotide transporter), member 9

Supplementary Table 7: Characterization of novel SNVs. The number of SNVs not found in KGP, dbSNP 142 and AGVP are shown. SAHGP_ALL include all 23 samples except the admixed Xhosa (XHD). The SEB includes 7 SOT, 7 XHS and the ZUS individual.

**Category**	**SAHGP_ALL (n=23)**	**SEB (n=15)**
Not in dbSNP	1306629	949654
Not in dbSNP and KGP	1276716	927781
Not in dbsSNP and KGP and AGVP	815404	545960

Supplementary Table 8: Genomic distribution of novel SNVs. (a) Selected genomic regions showing high enrichment of novel SNVs (b) The only region showing statistically significant enrichment of novel exonic SNVs.

**CHR**	**Start**	**End**	**Novel**	**Total**	**-log10(P)**	**Some of the Genes in the Regions**
(a) Novel SNV enirhced regions						
14	19000001	20000000	1032	3386	>300	*OR11H12, POTEG,POTEM*
21	9000001	10000000	1050	2988	>300	*TEKT4P, DQ579288*
22	16000001	17000000	830	3677	284,14	*DQ573684,DQ587539*
8	7000001	8000000	689	2634	277,047	*DEFA5, DEFB103A,SPAG11B, FAM66B,ZNF705G*
4	49000001	50000000	829	4147	245,44	*CWH43, DQ590126*
9	43000001	44000000	559	2166	221,901	*ANKRD20A3, SPATA31A6, FAM95B*
(b) Novel Exonic SNV enriched region						
**CHR**	**Start**	**End**	**Novel**	**Total**	**-log$_{10}$(P)**	**Some of the Genes in the Region**
16	2000001	3000000	38	329	5,98	*RPL3L,NDUFB10,RNF151,NOXO1,GFER,SLC9A3R2,TSC2,PKD1,BRICD5,ECI1,RNPS1,TBC1D24,AMDHD2,KCTD5,SRRM2,PRSS33,PRSS21,FLYWCH2,FLYWCH1*

Supplementary Table 9: SNV density differences. Some of the genomic regions (25 kb window) showing high SNV density differences among African populations are shown.

Genomic region (Chr_Start_End)	SEB	ZUL	YRI	LWK	Genes in the regions	Description of genes
1_12875001_12900000	296	243	422	447	*PRAMEF11*	PRAMEF11 (PRAME Family Member 11) is a Protein Coding gene. GO annotations related to this gene include *retinoic acid receptor binding*.
1_248800001_248825000	306	281	464	506	*OR2T35,OR2T27*	Olfactory receptor family genes
2_89150001_89175000	149	204	640	572	*IGKC,IGKJ5,IGKJ4,IGKJ3,IGKJ2,IGKJ1*	Immunoglobulin family genes
2_89250001_89275000	221	285	171	176	*IGKV1-6*	IGKV1-6 (Immunoglobulin Kappa Variable 1-6) is a Protein Coding gene. Diseases associated with IGKV1-6 include haemophilus infuenzae
4_8950001_8975000	326	314	509	545	*UNC93B8*	Pseudogene
6_32350001_32375000	317	436	687	873	*HCG23,BTNL2*	BTNL2 (Butyrophilin-Like 2) is a Protein Coding gene. Diseases associated with BTNL2 include sarcoidosis 2 and sarcoidosis 1./ HCG23 (HLA Complex Group 23 (Non-Protein Coding)) is an RNA Gene, and is affiliated with the lncRNA class.
12_8375001_8400000	323	302	461	446	*ALG1L10P,FAM86FP*	Pseudogene
12_9550001_9575000	252	254	430	494	*DDX12P*	Pseudogene
14_105400001_105425000	325	372	678	724	*AHNAK2*	AHNAK Nucleoprotein 2) is a Protein Coding gene. Diseases associated with AHNAK2 include skeletal muscle regenaration
17_43900001_43925000	225	299	145	284	*SPPL2C*	(Signal Peptide Peptidase Like 2C) is a Protein Coding gene. GO annotations related to this gene include *protein homodimerization activity* and *aspartic-type endopeptidase activity*.
17_43950001_43975000	**206**	**283**	126	**300**	*MAPT*	MAPT transcripts are differentially expressed in the nervous system, depending on stage of neuronal maturation and neuron type. MAPT gene mutations have been associated with several neurodegenerative disorders such as Alzheimer's disease, Pick's disease, frontotemporal dementia, cortico-basal degeneration and progressive supranuclear palsy.

Supplementary Table 10: Populations used in various analyses

Pop Code	Population Description	Number of Samples	Coverage/SNP chip	PCA and Structure	SNV density	f2 analysis	Population distances(FST)
**SAHGP WGS data**							
SOT	Sotho from South Africa	7	~50 X	✓			✓
XHS	Xhosa from South Africa	7		✓			✓
COL	South African Coloured from Western Cape, South Africa	8		✓		✓	✓
ZUS	South African Zulu from Soweto	1		✓			
XHD	Admixed Xhosa individual from South Africa (originally in XHS)	1		✓			
SEB	Sotho, Xhosa and Zulu from South Africa (SOT+XHS+ZUS)	15(7+7+1)			✓	✓	
**1000 Genomes Phase 3 WGS data**							
YRI	Yoruba in Ibadan, Nigeria	~100 (per population)	~2-4X	✓	✓	✓	✓
LWK	Luhya in Webuye, Kenya				✓	✓	✓
GWD	Gambian in Western Divisions in the Gambia					✓	
MSL	Mende in Sierra Leone					✓	
ESN	Esan in Nigeria					✓	
ASW	Americans of African Ancestry in SW USA					✓	
ACB	African Caribbeans in Barbados					✓	
JPT	Japanese in Tokyo, Japan					✓	
CHB	Han Chinese in Bejing, China			✓		✓	
CHS	Southern Han Chinese					✓	
CDX	Chinese Dai in Xishuangbanna, China					✓	
KHV	Kinh in Ho Chi Minh City, Vietnam					✓	
TSI	Toscani in Italia					✓	
FIN	Finnish in Finland					✓	
GBR	British in England and Scotland					✓	
IBS	Iberian Population in Spain					✓	
MXL	Mexican Ancestry from Los Angeles USA					✓	
PUR	Puerto Ricans from Puerto Rico					✓	
CLM	Colombians from Medellin, Colombia					✓	
PEL	Peruvians from Lima, Peru					✓	
GIH	Gujarati Indian from Houston, Texas			✓		✓	
PJL	Punjabi from Lahore, Pakistan					✓	
BEB	Bengali from Bangladesh			✓		✓	
STU	Sri Lankan Tamil from the UK					✓	

ITU	Indian Telugu from the UK					✓	
CEU	Utah Residents (CEPH) with Northern and Western European Ancestry			✓		✓	
**African Genome Variation Project WGS data (Gurdasani *et al.* 2015)**							
ZUL	Zulu from South Africa	~100 (per population)	4X	✓	✓		✓
BAG	Baganda from Uganda			✓			✓
**Singapore Sequencing Malay Project (Wong *et al.* 2013)**							
SSMP	Malay from Singapore	100	~30X	✓			
**Various Populations from Southern Africa (Schlebush *et al.* 2012)**							
**Pop Code**	**Population Description**	**Number of Samples**	**Coverage/SNP chip**	**PCA and Structure**	**SNV density**	**f2 analysis**	**Population distances(FST**
Ju/Õhoansi	Ju/Õhoansi	220 Individuals (~20 per population)	Illumina 2.5M chip	✓			✓
!Xuun	!Xuun			✓			✓
G|ui and G||ana	G|ui and G||ana			✓			✓
Nama	Nama			✓			✓
Khwe	Khwe			✓			✓
≠Khomani	≠Khomani			✓			✓
Karretjie	Karretjie			✓			✓
SWB	Herero			✓			✓
SEB2	Bantu-speakers			✓			✓
KS	All Khoesan (JUL+XUN+GGK+KHO+KAR+NAM+KHW)						
KSA	Northern and central Khoesan (JUL+XUN+GGK)			✓			
KSB	Southern Khoesan (KHO+KAR+NAM+KHW)			✓			
COLC	Coloured(Colesberg)			✓			
COLW	Coloured(Wellington)			✓			
**Black Southern African population (May *et al.* 2013)**							
**Pop Code**	**Population Description**	**Number of Samples**	**Coverage/SNP chip**	**PCA and Structure**	**SNV density**	**f2 analysis**	**Population distances(FST)**
SEB3	Black South Africans from Soweto	94 Individuals	Illumina 5M chip	✓			

Supplementary Table 11: F_{ST} analysis. Selected regions showing extreme allele frequency differences between SOT and XHS identified using a 25kb sliding window scan.

Chr	Start	End	Av_F_{ST}	Associated Genes
1	149075001	149100000	0.31	*NBPF23*
1	149175001	149200000	0.32	*RNVU1-17*
1	231400001	231425000	0.30	*RNA5SP80*
2	74875001	74900000	0.32	*SEMA4F*
2	143850001	143875000	0.38	*MTND6P11,MTND5P24,MTND4P22,MTND3P9*
4	75225001	75250000	0.31	*EREG*
5	148475001	148500000	0.39	*RN7SKP145*
9	77100001	77125000	0.35	*RORB*
6	118850001	118875000	0.30	*PLN*
6	127000001	127025000	0.33	*RPS4XP9*
9	138700001	138725000	0.31	*CAMSAP1*
10	23475001	23500000	0.37	*PTF1A,C10orf115*
11	55400001	55425000	0.43	*OR4P4,OR4S2*
11	55425001	55450000	0.36	*OR4C6,OR4V1P*
14	62225001	62250000	0.30	*SNAPC1*
14	90200001	90225000	0.32	*CHORDC2P*
18	12650001	12675000	0.30	*PSMG2,CEP76*
20	58525001	58550000	0.38	*CDH26*
22	30750001	30775000	0.32	*CCDC157,RNF215*

Supplementary Table 12: Populations and Super-populations included in Runs of Homozygosity (ROH) analysis. All the data were based on Omni 2.5M SNP chip, genotyped in three different studies (AGVP, Schlebusch *et al.* 2012 and SAHGP). NS denotes the number of samples.

Super-populations	NS	Constituent Population	NS	Data Source
West African Niger-Congo speakers (WNC)	319	Mandinka	88	AGVP
		Jola	79	AGVP
		Fula	74	AGVP
		Wolof	78	AGVP
Central West African Niger Congo Speakers (CNC)	299	YRI	100	AGVP
		Ga-Adangbe	100	AGVP
		Igbo	99	AGVP
Southern African Niger Congo Speakers (SNC) (These are Bantu speakers. The Bantu languages are an important sub-class of the Niger-Congo languages, and most South African's home language is a Bantu language such as isiZulu, isiXhosa, or seSotho.)	234	Zulu	100	AGVP
		Sotho	86	AGVP
		SOT	8	SAHGP
		XHS	8	SAHGP
		SEB2	20	Schlebusch et al. 2012
		SWB	12	Schlebusch et al. 2012
Southern African Khoesan (SKS). Khoesan languages are not related to Niger-Congo languages.	148	GGK	15	Schlebusch et al. 2012
		JUO	18	Schlebusch et al. 2012
		KAR	20	Schlebusch et al. 2012
		KHO	39	Schlebusch et al. 2012
		KHW	17	Schlebusch et al. 2012
		NAM	20	Schlebusch et al. 2012
		XUN	19	Schlebusch et al. 2012
Southern African Admixed populations (SAd)	48	COLC	20	Schlebusch et al. 2012
		COLW	20	Schlebusch et al. 2012
		COL	8	SAHGP
East African Nilo Saharan speakers (ENS)	100	Kalenjin	100	AGVP
East African Niger Congo Speakers (ENC)	470	Baganda	100	AGVP
		Banyarwanda	100	AGVP
		Barundi	97	AGVP
		Kikuyu	99	AGVP
		LWK	74	AGVP
East African Afro-Asiatic Speakers (EAA)	107	Amhara	42	AGVP
		Oromo	26	AGVP

		Somali	39	AGVP
African-American admixed populations (AAd)	121	ACB	72	AGVP
		ASW	49	AGVP
East Asian (EAS)	459	CDX	83	AGVP
		CHS	98	AGVP
		CHB	86	AGVP
		JPT	96	AGVP
		KHV	96	AGVP
South Asians(SAS)	95	GIH	95	AGVP
European(EUR)	474	CEU	95	AGVP
		GBR	91	AGVP
		IBS	99	AGVP
		FIN	97	AGVP
		TSI	92	AGVP
American(AMR)	234	CLM	65	AGVP
		MXL	47	AGVP
		PEL	50	AGVP
		PUR	72	AGVP

Supplementary Notes

Supplementary Note 1: Sample collection, Sequencing and Variant Calling

Sample collection and sequencing

Participants from 4 different ethnolinguistic groups were enrolled. Self-identification was considered as the basis of the ethnolinguistic group membership in the recruitment process. The first group consisting of individuals of mixed ancestry (referred to as Coloured in the South African context) was recruited from the Western Cape (COL). The second group included Sotho-speakers and they were recruited from in and around the town of Ventersburg in the Free State Province (SOT). The third group consists of Xhosa-speakers (Nguni language) from Port Elizabeth in the Eastern Cape Province (XHS). In addition, a Zulu-speaker (Nguni language) was recruited from Johannesburg (ZUS). The DNA samples were normalized to ~60ng per µl and ~5µg DNA was submitted to the Illumina Service Centre for sequencing on the Illumina HiSeq 2000 instrument (~100bp paired-end reads, ~314bp insert size).

1.1. Whole Genome Alignment and Processing

Initial analysis of the raw read data was conducted using the Isaac Analysis Pipeline version 2.0.2 and the reads were aligned to NCBI 37 (hg19) of the human genome reference sequence using the Isaac Alignment Software[1]. The Isaac Aligner identifies the complete set of relevant candidate mapping positions using a 32-mer seed-based search and then selects the best mapping among all candidates based on the optimal alignment score using a Bayesian model. During the mapping selection phase, low quality 3' ends and adaptor sequences were trimmed. Following the optimal mapping of reads, duplicates were identified and marked and realignment around indels performed. The final output from the Isaac Aligner[1] was a sorted duplicate marked, indel realigned BAM file. Since we were using GATK HaplotypeCaller version 3.2.2[2] to recall the variants, which deals with indel realignment more accurately in high coverage data (http://gatkforums.broadinstitute.org/gatk/discussion/7847/changing-workflows-around-calling-snps-and-indels), we did not repeat the indel realignment step. Prior to variant calling, Base Quality Score Recalibration (BQSR) was conducted on the BAM files using GATK, to correct for any systematic bias in the base quality scores generated during sequencing. The flagstats command in SAMtools[3] (SAMTOOLS REF) version 1.1-26-g29b0367 was used to generate metrics to assess the quality of the alignments. The total number of reads that passed quality control, and the number of duplicate and mapped reads per sample, are shown in **Supplementary Table 1a.** Based on these metrics we did not flag any samples for further downstream inspection as none of the samples fell outside of our defined quality thresholds: Fraction of reads aligned to reference < 90%, fraction of duplicates > 5%, percentage of paired mapped reads < 90%.

1.2. Single Nucleotide Variant Calling

Single nucleotide variant calling was performed on all samples using the Isaac variant caller. The Isaac Variant Caller uses a mismatch density filter to avoid calling variants in regions where there is an unexpectedly high number of disagreements with the reference. This approach is used to minimise the number of false positive variants called. In order to assess the accuracy of the variants called by the Isaac Variant Caller, we re-called variants at two independent sites using HaplotypeCaller in version 3.2-2 of GATK[2]. The HaplotypeCaller algorithm first identifies potential variants in each individual sample and then performs joint genotyping of SNVs and indels, leveraging information from all samples in the cohort to improve the sensitivity and precision of both variant and reference calls. The variant calling was conducted independently at the University of the Witwatersrand (Wits) and the University of Pretoria (UP) using the same GATK pipeline with different parameters. The Wits site conducted the variant calling using GATK's suggested best practices parameters, whilst UP used more stringent variant calling parameters as shown below:

Wits

```
GenomeAnalysisTK.jar \
-nct 4
-T HaplotypeCaller  \
-R ucsc.hg19.fasta \
-I SAMPLE.bam \
--emitRefConfidence GVCF \
--variant_index_type LINEAR \
--variant_index_parameter 128000 \
--dbsnp dbsnp_138.hg19.vcf \
-stand_call_conf 30 \
-stand_emit_conf 30 \
-o samplename.g.vcf
```

UP

```
GenomeAnalysisTK.jar \
-nct 8 \
-T HaplotypeCaller \
-R ucsc.hg19.fasta \
-I SAMPLE.bam \
--emitRefConfidence GVCF \
--variant_index_type LINEAR \
--variant_index_parameter 128000 \
--dbsnp dbsnp_138.hg19.vcf  \
-stand_call_conf 50.0 \
-stand_emit_conf 10.0 \
-o samplename.g.vcf
```

The variant calling per sample was followed by the joint genotyping step, whereby the genotypes for each sample were jointly called for each of the three groups independently; namely the COL, SOT and XHS (including the ZUS individual). An example of the script for the joint genotyping step of the XHS individuals is shown below:

```
GenomeAnalysisTK.jar \
-T GenotypeGVCFs \
-R ucsc.hg19.fasta \
--dbsnp dbsnp_138.hg19.vcf \
--variant A03.g.vcf \
--variant B03.g.vcf \
--variant C03.g.vcf \
--variant D03.g.vcf \
--variant E03.g.vcf \
--variant F03.g.vcf \
--variant G03.g.vcf \
--variant H03.g.vcf \
-o xhosa_gatk.vcf
```

1.3 Variant Filtering

The three SNV variant calling datasets were filtered to remove any false positive SNVs called due to sequencing or alignment errors. The variants called by the Isaac Variant Caller were filtered based on the following features:

IndelConflict - Locus in region with conflicting indel calls
SiteConflict - Site genotype conflicts with proximal indel calls, typically a heterozygous SNV call made inside of a heterozygous deletion
LowGQX - Locus Genotype Quality assuming variant position (GQX) is less than 30 or absent
HighDPFRRatio - The fraction of base calls filtered out at a site is greater than 0.4
HighSNVSB - SNV strand bias value (SNVSB) exceeds 10
HighDepth - Locus depth is greater than three times the mean chromosome depth

For the GATK variant calling datasets we used the GATK Variant Quality Score Recalibration (VQSR) to filter out possible spurious SNVs. VQSR uses a machine learning approach to learn a distribution model to describe the cluster boundaries of likely true variants from a set of training variants (e.g. known SNPs from HapMap) based on various SNV annotations. The algorithm generates two Gaussian mixture models, one based on sites known to be truly polymorphic and the second based on sites that are known to be possible true negatives.

For both GATK variant calling datasets we used the following training datasets and annotation features:

Training datasets

- HapMap version3.3 (prior 15.0)
- 1000Genomes_Omni2.5M (prior 12.0)
- 1000Genomes_phase1 (prior 10.0)
- dbSNP_138 (prior 2.0)

Annotations/Features

- QD – Quality by depth
- MQRankSum – Mapping quality rank sum test
- ReadPosRankSum – Read position rank sum test

- FS – Fisher's test on strand bias
- DP – Depth of coverage
- SOR – Strand odds ratio
- MQ – Mapping quality

Following the training of the models, the GATK Apply Recalibration was used to apply the models to all sites in both datasets using a tranche of 99.5, based on the theoretical ROC curves using the transition/transversion (Ti/Tv) ratio of novel SNVs as a measure of specificity.

1.4. Generation of final SNV dataset

We examined the concordance of the filtered GATK variant call sets from the two independent sites and the Isaac variant call set from Illumina and found a significant amount of overlap of over 97% of SNVs called between the three approaches (**Supplementary Figure 1c)**. Since all three datasets were independently called and filtered, we decided to generate a combined dataset based on the intersect of all three approaches in order to move forward with a high quality robust set of SNVs. Due to the modest sample size, we also assessed the Ti/Tv ratio in the final combined dataset as a function of minor allele counts to ensure that the variants identified in downstream analysis were unlikely to be false positives **(Supplementary Figure 1b, Supplementary Table 2b)**.

1.5 SNP array data and concordance

Each of the 24 samples was also genotyped on the Illumina Omni 2.5 genotyping array. We observed more than 99.5% concordance between the genotypes called by sequencing and chip-based genotyping, which validated the sequencing and variant calling methods employed.

1.6 Coverage

We analysed the difference in coverage between samples as well as within various genomic regions. The coverage achieved for each sample is summarized in **Supplementary Table 1b.** We did not detect any bias in the depth of coverage within genomic regions. Although we could observe the overall coverage in Xhosa (XHS) to be lower than the other two populations a t-test based evaluation showed the differences to be statistically significant only in the XHS-COL but not in the other two comparisons (SOT-COL and XHS-COL). Therefore, the observed differences do not appear to be related to demographic history or ancestry. The number of SNVs initially called and recalled using the steps mentioned above are provided in **Supplementary Table 2.** Around 16.3M unique SNVs were detected in the four populations, of which, about 6M were singletons (**Figure 1c).**

1.7 Site Frequency Spectrum (SFS)

As an additional measure to evaluate the quality of sequencing, we analyzed the site frequency spectrum in the three study populations and compared them to the SFS observed in the same number of randomly selected individuals from the African Genome Variation Project (AGVP) Zulu and the1000 Genomes Project (KGP) YRI, ASW, CEU populations[4,5]. We observed an overall agreement of the SFS in the SOT and XHS with the SFS in African populations from the two datasets. Moreover, a slightly higher proportion of singletons were observed in the SOT and XHS in

comparison to the COL as well as to random sample sets of the same size drawn from various African, Non-African and African-admixed populations (**Figure 1f**). The variation in SFS between populations is well known and studies have shown African populations to harbour more rare variants compared to non-African populations[6,7]. The relatively lower rate of singletons in the COL (as well as ASW, the other admixed population) can be explained on the basis of this.

While the differences in the SFS from the random African populations compared to SOT and XHS might lead to the hypothesis for an elevated singleton rate due to Khoesan admixture in the SOT and XHS, these differences could as well have been caused by differences in the sequencing depths of the two datasets. Han and colleagues' investigation[8] on SFS and its relationship to sequencing depth and the variant calling approach employed has shown that estimating genotypes by pooling individuals in a sample set (multisample calling) in low coverage data (as used in KGP and the AGVP) results in underestimation of the number of rare variants, which aligns with our observation. However, the slight enrichment of singletons in the SFS of the Zulu in comparison to YRI, both of which are low-coverage datasets suggests that observed differences in addition to technical differences might also be due to geography/admixture based differences (**Figure 1f**). High-coverage sequence datasets of comparable sample sizes from other African populations would enable us to investigate the observed differences in follow up studies.

1.8 Indels and copy number variant calling

Indels and copy number variants were called using the Isaac variant caller[1] software according to the Illumina pipeline. The indels and copy number variants called are summarized in **Supplementary Table 3**.

An average of 70 CNVs were identified per individual. For all the CNVs that were annotated by Illumina to overlap a gene region, we searched Ensembl to discover which CNVs completely contain at least one transcript of one gene. For each such gene, we found how many individuals contained a transcript of that gene. There are 121 CNVs which were found to contain at least one transcript of an Ensembl gene.

Supplementary Note 2: Other datasets used in the study

To contextualize the genotype data generated in the current study we included WGS sequence and genotyping-chip based data from various sources including the, AGVP[4], KGP[5], Malays from the Singapore Sequencing Malay Project (SSMP)[9], Black South Africans from Soweto (SEB3)[10] and several populations from the study by Schlebusch *et al*[11], namely several Khoesan (KS) and Coloured groups (WCOL), southeastern Bantu-speakers (SEB) and southwestern Bantu-speakers (SWB). The details of the datasets and the analyses they were included in are detailed in **Supplementary Table 10.**

Preliminary analysis was done to choose appropriate proxy populations (for ancestral populations) for the population structure and admixture analyses. In the initial analysis, the inclusion of KGP data sets from regions west of Nigeria, and the inclusion of the ESN data did not appear to provide any explanatory power and therefore these populations were omitted form the analyses.

Since the South African Coloured population has a complex admixture, we particularly wanted to select the most appropriate proxy populations. Prior work has used Chinese populations (particularly CHB) and the Gujrati (GIH) populations from HapMap[12] data. Given the historical background of these populations neither is likely to be ideal as historical evidence suggests that most of the Asian ancestry of the Coloured population is likely to have come from the east coast of India and the Indonesian archipelago[13].

We had access to new public data sets that prior studies did not. As a proxy for the Southeast Asian ancestry, the SSMP data proved far superior to the Chinese data sets that previous studies have used. When we included both CHB and SSMP data, both the COL data from the current study and the Coloured individuals in the data of Schlebusch *et al* 2012[11] appear to have negligible Chinese ancestry (<1%), which is what we would expect based on historical records. As an aside, it would be interesting to investigate the Indonesian populations as they might prove to be more accurate proxies in comparison to the SSMP. Nevertheless, now that there are many good Southeast Asian data sets available, Chinese populations should no longer be used in population studies of South African Coloured populations.

We also ran preliminary experiments with GIH and BEB data sets. Based on this work, we believe that the BEB data set is the better proxy population to include. Although there is merit in doing a deeper study of South Asian/Indian ancestry in South African Coloured populations, given the degree of admixture we were exploring we decided to include only one of the South Asian/Indian populations in the current study.

Supplementary Note 3: Novel SNV identification and genomic distribution

To identify novel SNVs in the SAHGP Southern African Human Genome Programme (SAHGP) whole genome sequencing (WGS) data, we compared the SNVs identified in this study to three different datasets (dbSNP 142[14], KGP[5] and the AGVP[4] WGS datasets). Of the 16.3 million unique SNVs detected in the study, around 0.8 million were detected to be novel in comparison to the three datasets **(Supplementary Table 7**). Of these more than half a million novel SNVs were detected in the 15 SEB speakers (**Supplementary Table 7)**. The high proportion of novel SNVs in the data, in spite of the inclusion of 100 Zulu whole genomes from the AGVP[4], can be ascribed to the inherent genetic diversity in southern Africa as well as high coverage whole genome sequencing. In addition to identifying these novel SNVs, this study was able to validate around half a million rare SNVs that has only been observed in AGVP[4] study.

The frequency distribution of the novel SNVs in the dataset is summarized in **Figure 1f**. Due to small sample size of the present study, most of these SNVs, as expected were found to occur once and only about 100K novel SNVs were observed to occur more than once in the dataset.

The distribution of novel SNVs was also found to vary widely among the 24 individuals (**Supplementary Figure 3a**). The variation was found to be most prominent in the COL.

To study the distribution of novel SNVs across the genome, a sliding window based scanning approach was employed. The number of novel SNVs in each 1Mb genomic window was recorded. The observed occurrences were assigned a p-value by comparing it to the total occurrence in novel SNVs in the genome using a hypergeometric test. The distribution of novel SNVs across the genome was found to be clearly non-random (**Supplementary Fig. 3b).** Some of the regions showing strong enrichment of novel SNVs are summarized in **Supplementary Table 8**. However, when we performed a similar comparison with exonic novel SNVs the distribution was found to be more homogeneous and only a single region in chromosome 16 was found to show enrichment of novel exonic SNVs **(Supplementary Figure 3c**).

Finally, we studied the distribution of novel SNVs in various functional categories as defined by ANNOVAR[15]. The relative representation of novel SNVs in various functional categories (with respect to all SNVs detected in our study) and minor allele count (MAC) classes is shown in **Figure 1g.** We observed a slightly higher representation of splicing, stop-gain and stop loss SNVs and a slightly lower representation of non-synonymous SNVs among singletons (MAC=1). This trend of representation was observed in other MAC classes too **(Figure 1g**). The exception to this was stop-loss SNVs, which were not detected in any other MAC class.

Supplementary Note 4: SNV density comparisons

The analysis of SNV density across the genome in a previous study based on about 90 Malay genomes has identified regions of functional significance like the HLA to show significantly higher SNV densities in contrast to the rest of the genome[9]. However, whether the SNV densities and their enrichment patterns vary among populations has not been studied. To study the variation of SNV enrichment patterns within Africa, we have compared SNV densities in the YRI and LWK populations from the KGP and the two southern African populations (SEB from the current study and the Zulu from the AGVP). For this, we scanned the genome using 1Mb sliding windows (with no overlap) and computed the number of SNVs in each 1Mb region for each of the four populations. The empirical distribution of SNV densities thus obtained for each population was used to assign a rank score and p-value to the density level observed for each window in that population. A similar scan was also conducted using 25kb windows. This was done so as to identify possible individual genes, which corresponds to high-density levels, as an 1Mb window generally involves a number of genes and it is difficult to infer individual genes from such scans.

Though the quantitative relationship between number of samples from a population included in a study and the SNV densities observed has not been elucidated, it can be assumed that higher sample sizes will essentially result in higher SNV density estimates. Therefore, in contrast to about 100 samples from the KGP populations, only 15 samples in the SEB might result in differences in estimation of SNV densities. Moreover, the difference in sequencing depth among these studies might have also biased the density estimates. Therefore, to identify genomic regions, which show distinctive SNV density distribution in southern Africans, we considered only the regions in which both Zulu and SEB were found to show similar SNVs densities and also to vary strongly with both the YRI and LWK populations. The comparison of SNV density between South African versus other African populations identified many genomic regions of notable SNP density difference. Some of these regions have been summarized in **Supplementary Table 9**. The potential functions of these genes were inferred using the GeneCards database (http://www.genecards.org). One of the highest differences in SNV density was observed in the genomic region containing the *PRAMEF1* gene. The *PRAMEF1* gene has been shown to be preferentially expressed in melanoma and also thought to function in reproductive tissues during development. Two contiguous 25kb windows in chromosome 17 were observed to show high SNV density differences and were found to contain genes like *MAPT* (associated to neurodegenerative disorders) and *SPPL2C* (associated to androgenetic alopecia, and progressive supranuclear palsy). Further analysis of regions showing high SNV density differences can be expected to provide clues to adaptation to new environments and large-scale changes in genomic architecture due to admixture.

We noted that a significant proportion of regions which were found to have high SNV density differences were associated with pseudogenes. While it can be speculated that the non-functional nature of pseudogenes might enable them to tolerate higher SNV densities in certain populations, it would be important to follow up on the source of variation in SNV density in these pseudogenes.

Supplementary Note 5: Principal Component Analysis (PCA)

For PCA and population structure analyses the data sets were combined using the following procedures. The relevant data sets (detailed in **Supplementary Note 2 and Supplementary table 10**) were merged using PLINK v1.9[16]. Any tri-allelic SNVs or SNVs that could possibly be ambiguous when merged with other data sets (A/T and C/G) were removed. We further filtered SNVs to remove any SNVs with >0.8% missing calls and any individuals with >2% missingness. This created a set of 951209 SNVs and 985 individuals with an overall genotyping rate of 99.9%. This dataset was then pruned using PLINK v1.9[16] to select SNVs not in linkage disquilibirum (LD) with each other (in each window of 1000, no pair of SNVs has $r^2>0.15$; (PLINK flag --indep-pairwise 1000 50 0.15). This led to a set of 197279 SNVs. In preliminary analysis, we experimented with different QC and pruning parameters and the results we present are robust with respect to those analyses. Relatedness among individuals was estimated using Idendity by Descent (IBD) approach in PLINK v1.9[16] and no relationship was observed among individuals in the current study.

Figure 2(a) and (b), and **Supplementary figures 4(a) and 4(b) and 6(a-e)** show the various PCs. In most of the figures we only show KGP, SAHGP and Khoesan data in the figures. Note that for clarity we do not show all populations in all figures but the PCs were computed in all cases using all the populations described in **Supplementary Table 10**. The XHS, SOT and ZUS individuals all cluster close to the YRI and LWK as expected, although the PC seems to indicate some admixture from the Khoesan (which in turn appear to have distinct sub-populations).

A very interesting observation was that the SOT and XHS samples appear to cluster distinctly. Even though the sample sizes are small, this is confirmed by Eigenstrat's ANOVA analysis (p-value $<10^{-5}$)[17]. Given the recent population divergence this was somewhat unexpected[18]. **Supplementary Note 6** shows that there is a small but observable difference in admixture with Khoesan groups, which is not surprising given the geographical locations of the groups[11,19]. Thus, we hypothesize that the difference may also be caused by difference in patterns of admixture with Khoesan groups rather than only divergence.

Supplementary Note 6: Population structure analysis

After selection of comparative groups was made, the pruned data set described above was analysed using ADMIXTURE 1.3.0[21]. Forty independent runs were used for each *K*=2…10. The results were combined using CLUMPP[22]. Using ADMIXTURE's ability to estimate error in the population ancestry estimates using the --cv flag, cross-validation error estimation scores were computed as shown in **Supplementary Figure 7c**. The optimal value for *K* was 7, which is also the smallest *K* value for which no obvious anomalies can be seen.

The results show that for *K*>5, there are significant differences between the southern African populations and those from central and western Africa. In addition, consistent with the results of Gurdasani *et al.* 2015[4], there is significant admixture from Khoesan populations in XHS, SOT (and ZUS and XHD). A significant Khoesan admixture was also observed in the COL. The **Supplementary Figure 5c** shows average population

ancestry for *K*=7 of all the populations in our study. We use as short-hand for the column headings the population that was dominant for that column (so for example, the CEU and YRI are highly homogeneous). Thus, the column labeled SEB is not intended to mean the ancestral SEB column but rather should be read as representing the ancestral population which is dominant in the SEB population.

The SOT and XHS data are very similar in proportions but the percentage admixture from Khoesan groups is significantly higher in the SOT than in the XHS (Mann-Whitney U-test p-value=0.026). **Supplementary Figure 5d** examines this in more detail. It is an extract of the table of average ancestry contributions for *K*=10. We only show the XHS and SOT together with the Khoesan (KS) data broken into two groups (KSA and KSB). The northern KS groups were merged to generate the KSA and the southern KS groups were merged to generate the KSB (Please see **Supplementary Table 10 for details**). At this greater level of resolution, it appears that the admixture is more likely from the KSB group than the KSA group. In the Coloured population, there is a complicated pattern of admixture with at least 5 ancestral populations contributing greater than 5% and no ancestral population contributing more than 30%.

Supplementary Note 7: Regions of allele frequency differentiation between Sotho and Xhosa

We studied the variation in F_{ST} score/value across the genome to identify regions that show high F_{ST} variation between the SOT and XHS. This study was aimed at detecting particular functional or environmental/adaptive factors that might have contributed to the differences between the populations.

For this analysis the SOT-XHS F_{ST} estimates were obtained for each SNV in the WGS data using PLINK v1.9[16]. A sliding window of 25kb was used to scan the distribution of average F_{ST} scores, across the genome. As the distribution of number of SNVs in genomic windows was not uniform and also not all SNVs in a genomic window were present in both the populations, we considered only the windows that contained at least 10 SNVs in both the populations. Although this approach increases the confidence of average F_{ST} estimates, it excluded many genomic regions that show enrichment of population-specific SNVs, which might also be interesting from a population differentiation point of view. The top 0.005% 25kb windows, showing highest F_{ST} difference between the two populations were identified and studied further (**Supplementary Figure 8 and Supplementary Table 11).**

We repeated this analysis with two other window sizes, 100kb and 1000kb to verify whether the observed F_{ST} differences are also seen for larger genomic regions. Both the analyses were able to identify a number of windows of strong F_{ST} differences, suggesting that these differences can span longer genomic regions and are distributed non-randomly across the genome.

Supplementary Note 8: f_2 analysis

The analysis of f_2 variants is based on the premise that if a SNV is seen to occur twice across a sample set, it would preferably occur twice in the same population or be shared by populations showing recent historical connections[5,23]. In order to compare rare allele sharing between the SAHGP and the KGP dataset, we merged the two datasets and identified those variants which occur precisely twice in the merged dataset (f_2 variants). As the sample sizes in the two datasets were not uniform and an unbiased estimate of f_2 sharing was difficult, we focused on those f_2 variants, which occur at least once in one of the 15 SEB (SOT+XHS+ZUS). As expected from population history the majority of the f_2 variants (73%) were shared within the SAHGP individuals. The sharing pattern with KGP populations has been summarized **in Figure 3a** and shows the SEB to share f_2 variants with both East African and West African populations. However, the sharing with the East African populations was observed to be greater than that with the West African populations. It is also interesting to note that among the Western African populations, the SEB were found to share most SNVs with the African ancestry population from the Caribbean followed by Esan from Nigeria. The insights from f_2 analysis are often suggestive and it would be important to devise follow up studies aimed at identifying the Western African populations which are closest to the South African Bantu-speakers.

It is also important to note that with small sample sizes, it is often difficult to ascertain the rarity of a variant and some of the f_2 variants in our analysis might not be actual f_2 variants. Therefore, to test if the observed pattern was an artifact of small sample size we have also analyzed the overall SNV sharing patterns between the South African and other populations. The SNVs included in this analysis were chosen such that they were present in the SEB and in only one of the populations from the KGP dataset, irrespective of their frequencies in the two populations. The sharing pattern of these SNVs between SAHGP and other populations (**Supplementary Figure 9a**) was found to be similar to that of the f_2 variants, emphasizing the observed trend of higher SNV sharing between SAHGP and other East African populations and recent historical contact between these groups. The higher sharing is probably due to the sharing of a more recent common ancestor or more recent genetic contact between the Bantu-speaking populations from South and East Africa compared to that between the South and West African Bantu-speakers[19,24].

The analysis when repeated for COL showed a similar f_2 sharing pattern with KGP populations (**Figure 3b and Supplementary Figure 9b**). Although the population is known to be highly admixed and various non-African ancestries were detected in other analyses[13,25,26], none of these were reflected in the f_2 analysis. However, this was not unexpected as the f_2 analysis included in the KGP study[5] also did not report second or subsequent ancestries in well-known admixed populations such as ACB ASW and MXL. It needs to be noted that the f_2 analysis is a method for identifying genetic relatedness between populations and should not be used for the purpose of detecting or quantifying admixture. Moreover, the southern African hunter-gatherers, who are known to be one of the major ancestral groups for the COL are not represented in the KGP dataset and therefore, the hunter-gatherer ancestry was not detectable in the present study design.

To extend this analysis we also studied the distribution of variants that were observed in the SAHGP populations and only one of the five KGP super populations/continental populations (East Asians (EAS), South Asians (SAS), Europeans (EUR), Americans (AMR) and Africans (AFR)). The distribution of such continent-specific SNVs from the KGP dataset in the SEB and COL is depicted in **Supplementary Figure 9c**. The analysis was able to identify African ancestry to be the predominant ancestry in both the groups but failed to reflect the admixture in the COL, reiterating that SNV-sharing based methods have limited capacity to detect complex admixtures.

Supplementary Note 9: F_{ST} based analysis of Khoesan affinities

The analysis of the fixation index (F_{ST}), at the whole genome level, provides an estimate of the genetic distance between any two populations and has been used extensively in inferring relationships between a set of populations[4,27]. We investigated the relationship between the South African populations in our datasets and two distinct sets of populations known to be related to them: the Niger-Congo (NC) speaking groups (from South, West and East Africa) and the Khoesan populations from southern Africa. For this analysis, a merged dataset consisting of data from the SAHGP, AGVP and Schlebusch *et al.* 2012 studies[4,11] was generated. The Weir and Cockerham's (WC) F_{ST}[28] estimate was computed between the SAHGP and other groups using PLINK v1.9[16]. Though there are various other estimates for F_{ST} and the values obtained using different estimates have been shown to vary significantly, we expected that even if the absolute values differ between estimates, the WC estimate would be able to capture the variation in relative distances between the studies groups.

The distribution of the KS populations used in the study as inferred from Schlebusch *et al.* 2012[11] is shown in **Figure 3c**. **Figure 3d** summarizes F_{ST} based estimates for the relationship between the three SAHGP populations and various southern African KS groups, suggesting the possible source of KS ancestry in these groups. In contrast to SOT, the XHS showed higher estimates of distance with respect to all the Khoesan groups, indicating possibly greater Khoesan genetic contribution in SOT compared to the XHS. Among the KS populations included in our analysis the Khwe was found to be closest to both SOT and XHS populations, whereas the Nama was found to be closest to the COL population according to F_{ST} estimates. The Ju/'hoansi followed by the !Xun were found to be most distant to all three groups. However, it is important to bear in mind that the admixture between the Bantu-speakers and KS has been bidirectional and the proximities of populations such as Khwe and SOT/XHS might also be strongly influenced by higher Bantu ancestry in these populations[19,24].

As all three populations sequenced in our study have some degree of KS admixture, there was a possibility that the difference in the levels of admixture could have affected the F_{ST} estimates between the study populations and various KS populations. To reduce the effect of admixture on the F_{ST} estimates we employed an approach similar to Gurdasani *et al.* 2015[4], in which we identified and masked the non-NC ancestry and computed F_{ST} for the NC only regions of the genome. The local ancestry detection tool, PCAdmix was used[29] with Ju/'hoansi (as the proxy for the KS), YRI (as the proxy for NC) and CEU, (as the proxy non-African) as three ancestral populations to identify 20 SNV genomic segments that show >80% NC ancestry in

the SEB2 from the Schlebusch *et al.* 2012 dataset[11]. Based on the local ancestry estimates, the non-NC regions were masked and F_{ST} between the three study populations and various KS groups were recalculated. The results summarized in **Supplementary Figure 10a** show the estimates of genetic distance between SOT, XHS, COL and various KS groups to be similar to that observed in **Figure 3d**. The XHS, as expected from its slightly higher NC ancestry, here shows lower genetic distances as we are visualizing the NC ancestry. The results, therefore, indicate the distance estimates to be similar for both the NC and the non-NC ancestry components.

The WC estimate for F_{ST} among the SOT and XHS and other Bantu-speaking populations from various parts of Africa has been summarized in **Supplementary Figure 10b**. Both SOT and XHO show similar distances with respect to other Bantu speaking populations. The SOT, however, showed slightly higher distance estimates throughout the comparisons. As expected, the southern African groups, the southeastern Bantu-speakers (SEB2) and the AGVP- Zulu (ZUL) were closest to the SOT and XHS. The Baganda, followed by LWK, were found to be the next most closely related populations, an observation that is also supported by the known pattern of Bantu-migration. Interestingly, in spite of relative geographic proximity the SOT and XHS were found to be most distant to southwestern Bantu-speakers (SEB) among all Bantu-speakers[19]. The distance between the SEB and SWB, perhaps reflect a relatively old divergence or high level of differential admixture in the two groups.

Supplementary Note 10: Analysis of Runs of Homozygosity

The study of runs of homozygosity (ROH) provided insights into population demographic history and also into the levels of admixture in a population[27,30,31]. Elevated levels of ROH have been suggested to correspond to background parental relatedness, often indicating a small population size or isolation of the ancestral population[27,30,31]. To identify possible distinctive features in the demographic history of the southern African populations, we compared the ROH in 23 individuals from the SAHGP study to other African and non-African populations from the AGVP dataset and various hunter-gatherer populations from the Schlebusch *et al.* 2012 datasets[4,11].

The three datasets, each genotyped on Illumina Omni 2.5M SNP chip, were merged using PLINK v1.9[16] (**Supplementary Table 12**). An overall QC was performed on the merged data and SNVs with missingness greater than 0.05 and individuals with missingness greater than 0.05, were removed. We also excluded SNVs showing extreme deviations from HWE (p-value<1×10^{-7}) from the data. To correct for possible ascertainment bias, SNVs with frequency lower than 0.01 in any of the super populations were removed (**Supplementary Table 12**). This resulted in a dataset containing around 500K SNVs (total genotyping rate in this dataset was 0.999182). Total ROH length and Number of ROH segments were estimated using PLINK v1.9[16]. By default, in PLINK v1.9[16] only runs of homozygosity containing at least 100 SNVs, and of total length ≥ 1000 kb are noted. Therefore, to identify shorter ROH segments we performed an additional analysis with the ROH window size set to 500kb. The scanning window hit was allowed to contain at most 1 heterozygous call and 5 missing calls. The Mann-Whitney U test was used to test differences between the total lengths of ROH distribution in population and super population pairs.

In addition to population level comparisons we also compared total ROH length distribution within various population groups or super-populations (**Figure 4a, Supplementary Table 12 and Supplementary Figure 11**). The comparison of total ROH length between African and non-African super-populations shows, as observed in a previous study[11], a considerable variation of ROH length among different African populations and super-populations (**Figure 4**, **Supplementary Figure 11**).

The lowest total ROH lengths were observed in the admixed populations from Western (ASW, ACB) and Southern Africa (COL). We found a considerable amount of ROH estimates variation within the NC-speaking groups from West, Central-West, East and South Africa. Among these the highest total ROH were detected in the South African Bantu-speaking populations, with the exception to Jola from West Africa and Baganda from East Africa, which showed comparable length and frequencies. Schlebusch and colleagues reported a similar cline in ROH distribution[11]. However, as the representation of the Bantu-speakers was much smaller in the dataset used in their study, these observations could have been due to individual level variations and not a population level phenomenon. The replication of the same trend in NC-speakers, in the present study, based on hundreds of samples confirms the observed trend. (**Figure 4, Supplementary Figure 11)**. The relatively higher ROH estimates for all the SEB suggest some distinctive demographic event/feature in southern Africa. In view of the relatively recent hunter-gatherer admixture in southern African populations [4,11], which should have effected a considerable reduction of ROH in these populations, the observation of relatively higher ROH estimates within this group is surprising and requires further investigation and explanation. The Eastern Nilo-Saharan populations had ROH lengths comparable to the Eastern NC-speakers. The highest total ROH lengths in the continent were observed in the KS and SWB from South Africa and Somali from East Africa. The high total ROH in Somali has not been reported and also needs to be explored further. The distribution of the number of ROH segments showed the same cline of variation among various populations (**Figure 4b**).

Supplementary Note 11: The Southern African Human Genome Programme (SAHGP) was launched at a meeting in January 2011. The following individuals participated in this meeting:

Soraya Bardien-Kruger, Sechaba Bareetseng, John Becker, Liza Bornman, Marietjie Botes, Jeff Chen, Alan Christoffels, Malcolm Collins, Marianne Cronje, Collet Dandara, Janita De Vries, Ames Dhai, Ben Durham, Fourie Joubert, Junaid Gamieldien, Jaco Greeff, Jaquie Greenberg, Anne Grobler, Scott Hazelhurst, Eileen Hoal, Lizette Jansen van Rensberg, Trefor Jenkins, Manjusha Joseph, Fourie Joubert, Amanda Krause, Derek Litthauer, Zané Lombard, Joe Molete, Marlo Moller, Nicola Mulder, Hugh Napier, Antonel Olckers, Thiri Padaychee, Michael Pepper, Bala Pillay, Tahir Pillay, Oliver Preisig, Raj Ramesar, Michèle Ramsay, Jasper Rees, Joanne Riley, Alison September, Thami Sithebe, Melodie Slabbert, Swasthi Soomaroo, Dawn Stephens, Magda Theron, Caroline Tiemessen, Nicki Tiffin, Stephen Tollman, Wayne Towers, Bruce Tshilamulele, Norma Tsotsi, Michael Urban, Lize van der Merwe, Carel van Heerden, Philip Venter, Louise Warnich.

Supplementary References

1. Raczy, C. *et al.* Isaac: ultra-fast whole-genome secondary analysis on Illumina sequencing platforms. *Bioinformatics* **29,** 2041–3 (2013).
2. McKenna, A. *et al.* The Genome Analysis Toolkit: a MapReduce framework for analyzing next-generation DNA sequencing data. *Genome Res.* **20,** 1297–303 (2010).
3. Li, H. *et al.* The Sequence Alignment/Map format and SAMtools. *Bioinformatics* **25,** 2078–2079 (2009).
4. Gurdasani, D. *et al.* The African Genome Variation Project shapes medical genetics in Africa. *Nature* **517,** 327–332 (2014).
5. Auton, A. *et al.* A global reference for human genetic variation. *Nature* **526,** 68–74 (2015).
6. Fu, W., Gittelman, R. M., Bamshad, M. J. & Akey, J. M. Characteristics of Neutral and Deleterious Protein-Coding Variation among Individuals and Populations. *Am. J. Hum. Genet.* **95,** 421–436 (2014).
7. Henn, B. M. *et al.* Distance from sub-Saharan Africa predicts mutational load in diverse human genomes. *Proc. Natl. Acad. Sci.* **113,** E440–E449 (2016).
8. Han, E., Sinsheimer, J. S. & Novembre, J. Fast and accurate site frequency spectrum estimation from low coverage sequence data. *Bioinformatics* **31,** 720–7 (2015).
9. Wong, L.-P. *et al.* Deep whole-genome sequencing of 100 southeast Asian Malays. *Am. J. Hum. Genet.* **92,** 52–66 (2013).
10. May, A. *et al.* Genetic diversity in black South Africans from Soweto. *BMC Genomics* **14,** 644 (2013).
11. Schlebusch, C. M. *et al.* Genomic variation in seven Khoe-San groups reveals adaptation and complex African history. *Science* **338,** 374–9 (2012).
12. The International HapMap Consortium. A haplotype map of the human genome. *Nature* **437,** 1299–320 (2005).
13. Daya, M. *et al.* A panel of ancestry informative markers for the complex five-way admixed South African coloured population. *PLoS One* **8,** e82224 (2013).
14. Sherry, S. T. *et al.* dbSNP: the NCBI database of genetic variation. *Nucleic Acids Res.* **29,** 308–311 (2001).
15. Wang, K., Li, M. & Hakonarson, H. ANNOVAR: functional annotation of genetic variants from high-throughput sequencing data. *Nucleic Acids Res.* **38,** e164 (2010).
16. Chang, C. C. *et al.* Second-generation PLINK: rising to the challenge of larger and richer datasets. *Gigascience* **4,** 7 (2015).
17. Price, A. L. *et al.* Principal components analysis corrects for stratification in genome-wide association studies. *Nat. Genet.* **38,** 904–909 (2006).
18. Lane, A. B. *et al.* Genetic substructure in South African Bantu-speakers: evidence from autosomal DNA and Y-chromosome studies. *Am. J. Phys. Anthropol.* **119,** 175–85 (2002).
19. Busby, G. B. *et al.* Admixture into and within sub-Saharan Africa. *Elife* **5,** (2016).
20. Bonner, P. & Segal, L. *Soweto: A History*. (Maskew Miller Longman, 1998).
21. Alexander, D. H., Novembre, J. & Lange, K. Fast model-based estimation of ancestry in unrelated individuals. *Genome Res.* **19,** 1655–64 (2009).
22. Jakobsson, M. & Rosenberg, N. A. CLUMPP: a cluster matching and permutation program for dealing with label switching and multimodality in

analysis of population structure. *Bioinformatics* **23,** 1801–6 (2007).
23. Mathieson, I. & McVean, G. Demography and the age of rare variants. *PLoS Genet.* **10,** e1004528 (2014).
24. Li, S., Schlebusch, C. & Jakobsson, M. Genetic variation reveals large-scale population expansion and migration during the expansion of Bantu-speaking peoples. *Proc. Biol. Sci.* **281,** (2014).
25. Patterson, N. *et al.* Genetic structure of a unique admixed population: implications for medical research. *Hum. Mol. Genet.* **19,** 411–9 (2010).
26. Chimusa, E. R. *et al.* Determining ancestry proportions in complex admixture scenarios in South Africa using a novel proxy ancestry selection method. *PLoS One* **8,** e73971 (2013).
27. The Genome of the Netherlands Consortium. Whole-genome sequence variation, population structure and demographic history of the Dutch population. *Nat. Genet.* **46,** 818–825 (2014).
28. Weir, B. S. & Cockerham, C. C. Estimating F-statistics for the Analysis of Population Structure. *Evolution (N. Y).* **38,** 1358–1370 (1984).
29. Brisbin, A. *et al.* PCAdmix: principal components-based assignment of ancestry along each chromosome in individuals with admixed ancestry from two or more populations. *Hum. Biol.* **84,** 343–64 (2012).
30. Kirin, M. *et al.* Genomic runs of homozygosity record population history and consanguinity. *PLoS One* **5,** e13996 (2010).
31. Pemberton, T. J. *et al.* Genomic patterns of homozygosity in worldwide human populations. *Am. J. Hum. Genet.* **91,** 275–92 (2012).
